# Head-to-head comparison of stress hyperglycemia ratio versus triglyceride-glucose index for predicting mortality in heart failure: a retrospective cohort study

**DOI:** 10.3389/fendo.2026.1782922

**Published:** 2026-02-12

**Authors:** Dongli Song, Shengnan Liu, Zuming Liu, Shuo Wu, Jiali Wang

**Affiliations:** Department of Emergency Medicine and Chest Pain Center, Shandong Provincial Clinical Research Center for Emergency and Critical Care Medicine, Qilu Hospital of Shandong University, Jinan, China

**Keywords:** heart failure, stress hyperglycemia ratio, triglyceride-glucose index, mortality, prognosis

## Abstract

**Background:**

Heart failure (HF) is a life-threatening clinical syndrome characterized by high incidence and mortality, leading to considerable global health and economic burdens. Stress-induced hyperglycemia ratio (SHR) and triglyceride-glucose (TyG) index, as emerging biomarkers reflecting glucose metabolism, are closely associated with poor prognosis in many diseases. However, it remains unclear which of these two indicators possesses superior association and predictive value for prognosis in critically ill patients with HF.

**Methods:**

A retrospective cohort study was conducted on critically ill HF patients, enrolled from the Medical Information Mart for Intensive Care IV (MIMIC-IV) version 3.1. The primary outcome was 180-day mortality, with 1-year (365-day), 90-day, and 30-day mortality as secondary outcomes. Baseline characteristics were compared between survivors and non-survivors. Cox regression, restricted cubic spline (RCS), Kaplan-Meier (K-M), and subgroup analyses were used to assess the association of SHR and TyG index with mortality. Discriminative performance of SHR versus TyG index was compared using ROC curves.

**Results:**

A total of 1,063 patients were enrolled. After adjusting for confounders, Cox regression analyses revealed that SHR was significantly associated with an increased risk of 180-day, 365-day, 90-day, and 30-day mortality, with hazard ratios (HRs) of 1.35, 1.26, 1.47, and 1.53, respectively. In contrast, TyG index was only associated with mortality risk at 180, 90, and 30 days (HRs: 1.20, 1.24, and 1.31, respectively), with no significant association observed at 1 year. Moreover, these associations were predominantly linear in nature. However, no statistically significant difference was observed in the predictive performance of SHR and TyG index for mortality at any time points (*P*>0.05).

**Conclusion:**

SHR and TyG index can be used as potential risk assessment tools for short-term (180-, 90-, and 30-day) mortality risk in critically ill HF patients, nevertheless, SHR is a more applicable and robust metabolic biomarker associated with 1-year mortality.

## 1 Introduction

Heart failure (HF) is a serious long-term clinical syndrome characterized by high morbidity and mortality, and it remains a leading cause of global hospitalization and death (1). Globally, the prevalence of HF is estimated at approximately 56 million cases (2). Despite significant improvements in survival rates due to advancements in pharmacotherapy and device-based treatments, the prognosis for HF patients remains poor, with a 5-year mortality rate exceeding 50% (3). Therefore, early identification of critically ill HF patients at high risk of mortality is of utmost importance.

Currently, the prognostic assessment of HF largely relies on biomarkers such as NT-proBNP and echocardiographic parameters. However, NT-proBNP levels can be influenced by various factors including renal function, age, and sex (4, 5), while the acquisition of echocardiographic data is dependent on specialized equipment and experienced operators. Concurrently, growing evidence indicates that critical illness is commonly accompanied by disorders of glucose metabolism (6), and that dysregulated glucose metabolism plays a key driving role in disease progression (7, 8). It can contribute to myocardial injury and cardiac dysfunction through multiple mechanisms, such as oxidative stress, inflammatory responses, and endothelial dysfunction (9, 10), and is closely linked to adverse outcomes in critically ill HF patients (9). Consequently, identifying biomarkers derived from glucose metabolism that can stratify mortality risk in these patients holds considerable clinical promise.

In recent years, the stress hyperglycemia ratio (SHR) and the triglyceride-glucose (TyG) index have gained significant attention as emerging biomarkers reflecting glucose metabolism status (11, 12). SHR is used to assess the true glycemic status of critically ill patients based on admission fasting blood glucose (FBG) and chronic blood glucose levels (13, 14); TyG index, calculated from triglycerides (TG) and FBG, serves as an indicator of insulin resistance (15). Both indicators are closely linked to a poor prognosis in cardiovascular disease (12, 16–18). However, it remains elusive which of these two biomarkers possesses superior predictive value for the prognosis of critically ill patients with HF.

Therefore, this study aimed to compare the association of SHR and TyG index with mortality risk in critically ill HF patients, in order to identify the superior predictor of all-cause mortality and facilitate early risk stratification for precise intervention.

## 2 Methods

### 2.1 Study design and population

This retrospective cohort study utilized data from the Medical Information Mart for Intensive Care IV (MIMIC-IV) version 3.1 database. This large, de-identified dataset contains information for patients admitted to the emergency department or intensive care units of Beth Israel Deaconess Medical Center in Boston, Massachusetts, between 2008 and 2022. The review committee granted waivers for patient informed consent and approved the data sharing plan. Prior to data extraction, the author (DLS) completed the required Collaborative Institutional Training Initiative (CITI) program, obtained database access permission, and performed the extraction (Record ID: 66402407). This study included patients who were diagnosed with HF, were admitted to the ICU for the first time, and were aged ≥18 years. The diagnosis of HF was based on the 9th and 10th editions of the International Classification of Diseases (ICD-code), as specified in **Supplementary Material 1**. Patients were excluded for any of the following reasons: (1) ICU length of stay<24h or missing data; (2) missing data for FBG, or HbA1c, or TG; (3) loss to follow-up.

### 2.2 Data extraction

We utilized PostgreSQL (version 17.0) and Navicat Premium Lite (version 17.2.9) to extract the following data from the first ICU admission: (1) baseline Characteristics: Age, sex, race, BMI, height, weight; (2) vital Signs: systolic blood pressure (SBP), diastolic blood pressure (DBP), heart rate, respiratory rate, Saturation of peripheral oxygen (SpO_2_), temperature; (3) Comorbidities: hypertension, diabetes, stroke, chronic kidney disease (CKD), acute kidney injury (AKI), atrial fibrillation (AF), chronic obstructive pulmonary disease (COPD), dyslipidemia, respiratory failure (RF), myocardial infarction (MI); (4) laboratory test indicators: blood cell count, including white blood cell (WBC), red blood cell (RBC), platelet, neutrophil, and lymphocyte; hemoglobin, troponin T, creatine kinase(CK), creatine kinase MB isoenzyme (CK-MB), C-reactive protein (CRP), albumin, blood urea nitrogen (BUN), creatinine (Cr), high-density lipoprotein cholesterol (HDL-C), low-density lipoprotein cholesterol (LDL-C), triglycerides (TG), fasting blood glucose (FBG), glycated hemoglobin (HbA1c), sodium, potassium, calcium, magnesium, and lactate; (5) echocardiographic measures: left ventricular ejection fraction (LVEF); (6) in-hospital Treatments: use of invasive/non-invasive mechanical ventilation (IMV), renal replacement therapy (RRT), and medications including norepinephrine (NE), dopamine, dobutamine, angiotensin-converting enzyme inhibitors (ACEI), angiotensin II receptor blockers (ARB), angiotensin receptor-neprilysin inhibitors (ARNI), diuretics, SGLT2 inhibitors, antiplatelet agents, anticoagulants, β-blockers, and statins; (7) others: ICU and total hospital length of stay, time of admission to ICU, date of death, sequential organ failure assessment (SOFA) score and simplified acute physiology score (SAPS) II score.

### 2.3 Calculation of SHR and TyG index, and outcomes

The TyG index was calculated using the formula: TyG index = Ln[(TG (mmol/L) × FPG (mmol/L))/2] (19), the SHR was calculated as SHR = [FPG (mmol/L)]/[1.59 × HbA1c (%) − 2.59] (13). The follow-up period commenced on the date of ICU admission. The primary outcome was 180-day mortality, and secondary outcomes were 1 year (365-day), 90-day, and 30-day mortality.

### 2.4 Statistical analysis

Statistical analyses were performed using R software (version 4.4.1, 2024-06-14; https://www.r-project.org/). The normality of continuous variables was assessed using the Jarque-Bera test. Normally distributed data are presented as mean ± standard deviation (M ± SD) and compared between two groups using Student’s t-test. Non-normally distributed data are presented as median and interquartile range [M (Q1, Q3)] and compared using the Mann-Whitney U test. Categorical variables are expressed as frequency (%) and compared between groups using the Chi-square test or Fisher’s exact test, as appropriate. Variables with more than 20% missing data were excluded from analysis, while those with 20% or less missing data were imputed using the random forest method. A two-tailed *P*-value < 0.05 was considered statistically significant.

Cox regression analysis was employed to assess the associations of SHR and TyG index with the risk of mortality in HF patients. Both SHR and TyG index were analyzed as continuous variables and as quartiles. Three models were constructed: model 1 was unadjusted; model 2 was adjusted for age, gender, and race; model 3 was adjusted for age, gender, race, AKI, diabetes, hypertension, stroke, CKD, AF, COPD, dyslipidemia, RF, MI. The variance inflation factor (VIF) was calculated, with a VIF < 5 indicating no significant multicollinearity among the variables. Trend tests (calculating the P for trend) was performed, and restricted cubic spline (RCS) curves were used to visualize the dose-response relationships of SHR and TyG index with the risk of mortality in patients with HF. The surv_cutpoint function was employed to dichotomize both SHR and TyG index based on their optimal cut-off values. Kaplan-Meier (K-M) curves were then generated to compare the relationship between the two groups (for each indicator) and mortality risk. ROC curves were plotted and the area under the curve (AUC) was calculated to evaluate the predictive performance of SHR and TyG index for mortality risk, both independently and in combination with the basic model. Subgroup analyses were performed based on prespecified variables: age, sex, hypertension, stroke, CKD, diabetes, respiratory failure, with HbA1c specifically for TyG index analysis and TG levels for SHR analysis.

## 3 Results

### 3.1 Baseline characteristics

**Figure 1:**
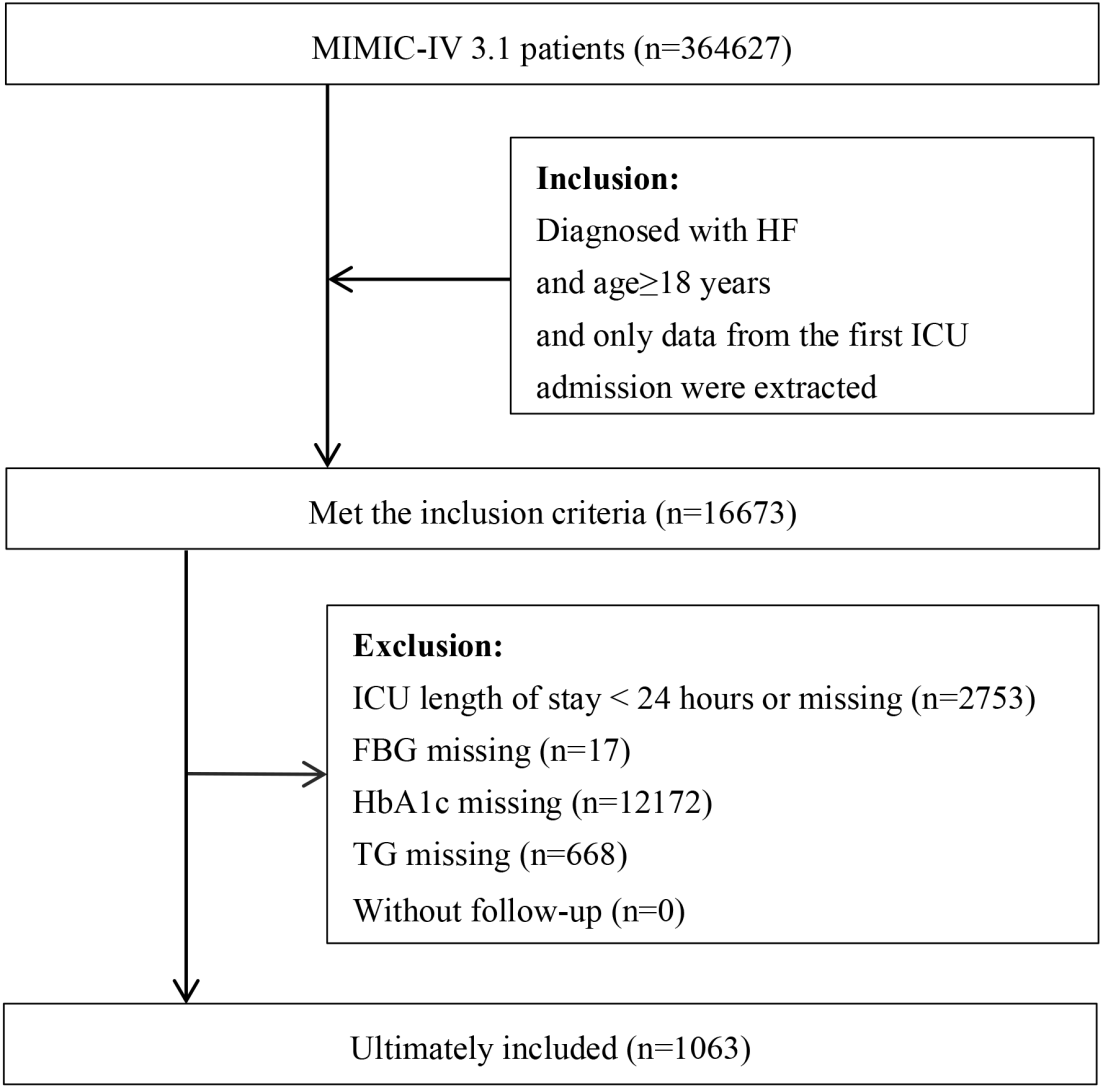
Flowchart of patient selection. MIMIC-IV, Medical Information Mart for Intensive Care-IV; HF, heart failure; FBG, fasting blood glucose; HbA1c, Hemoglobin A1c; TG, triglycerides.

This study ultimately included 1,063 critically ill HF patients. The patient selection flowchart is presented in **Figure 1**. The median age of the included cohort was 72.24 years, and 58.04% were female. Based on the primary outcome of 180-day mortality after ICU admission, patients were stratified into survivors (n=736) and non-survivors (n=327). Detailed comparisons of baseline characteristics are provided in **Table 1**. Compared with survivors, non-survivors were significantly older, had a lower proportion of white ethnicity and a higher proportion of unknown race, lower body weight, and higher SpO_2_. They also had a higher prevalence of comorbidities, including stroke, CKD, AKI, AF, COPD, and RF. Regarding laboratory findings, non-survivors exhibited higher WBC counts, BUN, and Cr levels, but lower RBC counts and LDL-C levels. In terms of in-hospital management, a higher proportion of non-survivors received NE, dobutamine, IMV, and RRT, while a lower proportion received β-blockers, ACEI/ARB/ARNI, antiplatelet agents, anticoagulants, statins, and non-IMV. Additionally, non-survivors had higher SOFA and SAPS II scores, alongside longer lengths of stay for both the hospital and ICU, as well as a higher value for the calculated SHR (all *P* < 0.05).

**Table 1:** Baseline characteristics by 180-day survival status in HF patients.

Variables	Total (n = 1063)	Survivor (n = 736)	Non-survivor (n = 327)	*P*
Age^a^, y	72.24 (61.69, 82.19)	69.32 (59.88, 80.52)	77.01 (67.31, 85.35)	**< 0.001**
Gender^b^				0.128
Female	617(58.04)	439 (59.65)	178 (54.43)	
Male	446 (41.96)	297 (40.35)	149 (45.57)	
Race^b^				**0.025**
White	619 (58.23)	439 (59.65)	180 (55.05)	
Black	97 (9.13)	71 (9.65)	26 (7.95)	
Asian	16 (1.51)	10 (1.36)	6 (1.83)	
Hispanic/Latino	28 (2.63)	22 (2.99)	6 (1.83)	
Other	42 (3.95)	34 (4.62)	8 (2.45)	
Unknown	261 (24.55)	160 (21.74)	101 (30.89)	
Weight^a^, Kg	80.00 (66.90, 96.12)	81.60 (68.54, 97.93)	75.80 (64.10, 92.03)	**< 0.001**
SBP^a^, mmHg	126 (109, 145)	126.5 (110.75, 145)	123 (107.5, 141)	0.092
DBP^a^, mmHg	73 (62, 86)	74 (62, 87)	72 (61, 85)	0.075
HR^a^, bpm	87 (73, 100.5)	87 (73, 100)	87 (73.5, 101.5)	0.526
RR^a^, bpm	20 (16, 23)	20 (16, 23)	20 (16, 24)	0.162
Spo_2_^a, %^	97 (94, 99)	97 (94, 99)	98 (94.5, 100)	**0.029**
Temperature^a^, °C	36.72 (36.44, 37.00)	36.72 (36.44, 37.06)	36.67 (36.44, 36.94)	0.084
Hypertension^b^				0.328
No	226 (21.26)	163 (22.15)	63 (19.27)	
Yes	837 (78.74)	573 (77.85)	264 (80.73)	
Diabetes^b^				0.292
No	638 (60.02)	450 (61.14)	188 (57.49)	
Yes	425 (39.98)	286 (38.86)	139 (42.51)	
Stroke^b^				**< 0.001**
No	599 (56.35)	461 (62.64)	138 (42.2)	
Yes	464 (43.65)	275 (37.36)	189 (57.8)	
CKD^b^				**< 0.001**
No	738 (69.43)	539 (73.23)	199 (60.86)	
Yes	325 (30.57)	197 (26.77)	128 (39.14)	
AKI^b^				**< 0.001**
No	157 (14.77)	127 (17.26)	30 (9.17)	
Yes	906 (85.23)	609 (82.74)	297 (90.83)	
MI^b^				0.857
No	654 (61.52)	451 (61.28)	203 (62.08)	
Yes	409 (38.48)	285 (38.72)	124 (37.92)	
AF^b^				**< 0**.**001**
No	519 (48.82)	397 (53.94)	122 (37.31)	
Yes	544 (51.18)	339 (46.06)	205 (62.69)	
Dyslipidemia^b^				0.089
No	503 (47.32)	335 (45.52)	168 (51.38)	
Yes	560 (52.68)	401 (54.48)	159 (48.62)	
COPD^b^				**0.048**
No	904 (85.04)	637 (86.55)	267 (81.65)	
Yes	159 (14.96)	99 (13.45)	60 (18.35)	
RF^b^				**< 0.001**
No	626 (58.89)	478 (64.95)	148 (45.26)	
Yes	437 (41.11)	258 (35.05)	179 (54.74)	
WBC^a^, K/μL	10.7 (8.1, 14.4)	10.5 (7.9, 14)	11.2 (8.35, 15.25)	**0.014**
RBC^c^, K/uL	4.00 ± 0.77	4.06 ± 0.75	3.87 ± 0.81	**< 0.001**
Platelet^a^, K/μL	208 (163, 270)	209.5 (165, 271.25)	200 (155.5, 268.5)	0.154
BUN^a^, mg/dl	22 (16, 35)	21 (15, 31)	27 (18, 46)	**< 0.001**
Cr^a^, mg/dl	1.1 (0.8, 1.6)	1.1 (0.8, 1.5)	1.3 (0.9, 1.8)	**< 0.001**
FBG^a^, mmol/l	7.83 (6.16, 10.77)	7.71 (6.05, 10.39)	8.21 (6.38, 11.27)	0.122
HbA1c^a^, %	5.9 (5.5, 6.8)	5.9 (5.5, 6.8)	5.9 (5.5, 6.7)	0.913
TG^a^, mmol/l	1.15 (0.86, 1.68)	1.15 (0.85, 1.69)	1.15 (0.87, 1.68)	0.786
HDL-C^a^, mg/dl	42 (33, 52)	42 (33.28, 53)	41.61 (32.16, 50)	0.264
LDL-C^a^, mg/dl	73.00(55, 97)	77.54 (55, 101.25)	66.14 (53, 88)	**< 0.001**
Sodium^a^, mEq/l	138 (135, 141)	138 (136, 141)	138 (135, 142)	0.508
Potassium^a^, mEq/l	4.1 (3.8, 4.6)	4.1 (3.8, 4.5)	4.2 (3.9, 4.7)	0.008
Calcium^a^, mg/dl	8.6 (8.2, 9.0)	8.7 (8.2, 9.1)	8.6 (8.1, 9.0)	0.146
Magnesium^a^, mg/dl	2 (1.8, 2.2)	2 (1.8, 2.1)	2 (1.8, 2.2)	0.155
NE^b^				**< 0.001**
No	817 (76.86)	601 (81.66)	216 (66.06)	
Yes	246 (23.14)	135 (18.34)	111 (33.94)	
Dopamine^b^				>0.999
No	1005 (94.54)	696 (94.57)	309 (94.50)	
Yes	58 (5.46)	40 (5.43)	18 (5.50)	
Dobutamine^b^				**0.005**
No	989 (93.04)	696 (94.57)	293 (89.60)	
Yes	74 (6.96)	40 (5.43)	34 (10.40)	
SGLT2i^b^				0.501
No	1046 (98.40)	726 (98.64)	320 (97.86)	
Yes	17 (1.60)	10 (1.36)	7 (2.14)	
β-blocker^b^				**< 0.001**
No	149 (14.02)	68 (9.24)	81 (24.77)	
Yes	914 (85.98)	668 (90.76)	246 (75.23)	
Diuretics^b^				0.217
No	211 (19.85)	154 (20.92)	57 (17.43)	
Yes	852 (80.15)	582 (79.08)	270 (82.57)	
ACEI/ARB/ARNI^b^				**< 0.001**
No	517 (48.64)	291 (39.54)	226 (69.11)	
Yes	546 (51.36)	445 (60.46)	101 (30.89)	
Antiplatelet^b^				**0.002**
No	221 (20.79)	134 (18.21)	87 (26.61)	
Yes	842 (79.21)	602 (81.79)	240 (73.39)	
Anticoagulation^b^				**0.010**
No	37 (3.48)	18 (2.45)	19 (5.81)	
Yes	1026 (96.52)	718 (97.55)	308 (94.19)	
Statins^b^				**0.005**
No	243 (22.86)	150 (20.38)	93 (28.44)	
Yes	820 (77.14)	586 (79.62)	234 (71.56)	
IMV^b^				**< 0.001**
No	670 (63.03)	509 (69.16)	161 (49.24)	
Yes	393 (36.97)	227 (30.84)	166 (50.76)	
non-IMV^b^				**< 0.001**
No	297 (27.94)	173 (23.51)	124 (37.92)	
Yes	766 (72.06)	563 (76.49)	203 (62.08)	
RRT^b^				**< 0.001**
No	955 (89.84)	687 (93.34)	268 (81.96)	
Yes	108 (10.16)	49 (6.66)	59 (18.04)	
SHR^a^, mmol/l	1.14 (0.91, 1.4)	1.12 (0.90, 1.38)	1.18 (0.93, 1.48)	**0.034**
TyG index^a^, mmol/l	1.55 (1.09, 2.08)	1.53 (1.08, 2.06)	1.57 (1.15, 2.12)	0.182
SOFA score^a^	4 (2, 6)	3 (2, 5)	5 (3, 8)	**< 0.001**
SAPS II score^a^	36 (29, 44)	33 (26, 40)	42 (35, 51)	**< 0.001**
LOS of hospital^a^, d	9.59 (5.46, 17.12)	9 (5.26, 15.86)	10.58 (5.71, 18.80)	**0.042**
LOS of ICU^a^, d	4.12 (2.24, 8.04)	3.71 (2.06, 6.82)	5.43 (3.04, 10.75)	**< 0.001**

^a^为M(*Q1,Q3*), ^b^为n(%), ^c^为M ± SD; HF, heart failure; SBP, systolic blood pressure; DBP, diastolic blood pressure; HR, heart rate; RR, respiratory rate; Spo_2_, saturation of peripheral oxygen; CKD, chronic kidney disease; AKI, acute kidney injury; MI, myocardial infarction; AF, atrial fibrillation; COPD, chronic obstructive pulmonary disease; RF, respiratory failure; WBC, white blood cell; RBC, red blood cell; BUN, blood urea nitrogen; Cr, creatinine; FBG, fasting blood glucose; HbA1c, hemoglobin A1c; TG, triglyceride; HDL-C, high-density lipoprotein cholesterol; LDL-C, low-density lipoprotein cholesterol; NE, norepinephrine; SGLT2i, sodium glucose cotransporter-2 inhibitor; ACEI/ARB/ARNI, angiotensin converting enzyme inhibitor/angiotensin receptor blocker/angiotensin receptor neprilysin inhibitor; IMV, invasive mechanical ventilator; non-IV, non-invasive ventilator; RRT, renal replacement therapy; SHR, stress hyperglycemia ratio; TyG, triglyceride-glucose; LOS, length of stay.

Bold font indicates that the *P*-value is < 0.05 (representing statistical significance).

### 3.2 Cox regression and RCS analyses of SHR and TyG index with 180-day mortality risk

**Figure 2:**
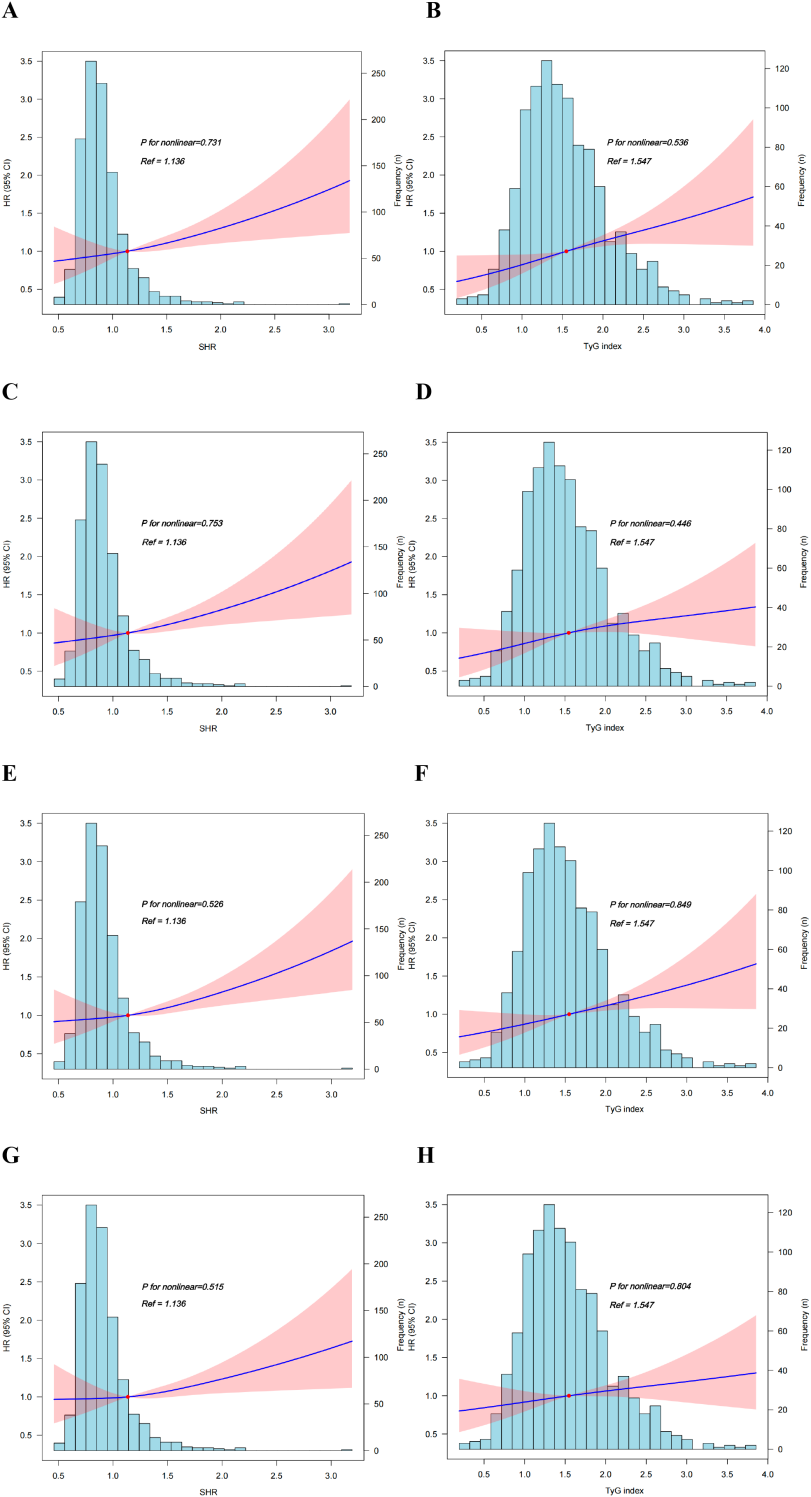
RCS curves of SHR and TyG index with 180-day and 1-year mortality [**A–D**, 180-day; **E–H**, 1-year. **(A, B, E, F)** adjusted for age, gender, race; **(C, D, G, H)**, adjusted for age, gender, race, hypertension, diabetes, stroke, dyslipidemia, myocardial infarction, atrial fibrillation, chronic kidney disease, acute kidney injury, chronic obstructive pulmonary disease, respiratory failure].

Analyzed as a continuous variable, SHR remained significantly associated with an increased 180-day mortality risk across all three models: Model 1: HR 1.34, 95% CI 1.13-1.59; Model 2: HR 1.44, 95% CI 1.21-1.72; Model 3: HR 1.35, 95% CI 1.12-1.64. Following this, patients were stratified into four quartiles based on SHR levels: T1 (<0.908; n=266), T2 (≥0.908 to <1.136; n=265), T3 (≥1.136 to <1.404; n=266), and T4 (≥1.404; n=266). The association was particularly pronounced in the highest quartile (T4), where SHR was associated with a markedly increased risk of 180-day mortality in all models: Model 1: HR 1.55, 95% CI 1.14-2.11; Model 2: HR 1.73, 95% CI 1.26-2.36; Model 3: HR 1.60, 95% CI 1.15-2.23. Subsequently, a test for trend was performed, which revealed a significant linear relationship between SHR and 180-day mortality risk across all models (Model 1: *P* for trend = 0.009; Model 2: *P* for trend = 0.001; Model 3: *P* for trend = 0.008) (**Table 2**). This linear dose-response relationship was visually confirmed by RCS analysis, which indicated no evidence of nonlinearity (Model 2: *P* for nonlinear = 0.731; Model 3: *P* for nonlinear = 0.753) (**Figure 2**). Furthermore, using the optimal cut-off value of SHR, patients were dichotomized into high (≥1.732) and low (<1.732) groups. The high group was associated with a significantly increased risk of 180-day mortality in all models (Model 1: HR 1.96, 95% CI 1.48-2.61; Model 2: HR 2.07, 95% CI 1.55-2.77; Model 3: HR 1.79, 95% CI 1.32-2.44) (**Table 2**).

**Table 2:** Associations of SHR and TyG index with mortality in HF patients.

Variables	Groups	180-day mortality			1-year mortality		
		Model 1	Model 2	Model 3	Model 1	Model 2	Model 3
		HR (95% CI) *P*-Value	HR (95% CI) *P*-Value	HR (95% CI) *P*-Value	HR (95% CI) *P*-Value	HR (95% CI) *P*-Value	HR (95% CI) *P*-Value
SHR	**Continuous**	1.34(1.13-1.59) **<0.001**	1.44(1.21-1.72) **<0.001**	1.35(1.12-1.64) **0.002**	1.26(1.06-1.49) **0.007**	1.35(1.13-1.60) **<0.001**	1.26(1.04-1.52) **0.018**
	Quartiles						
	T1(<0.908; n=266)	Ref	ref	Ref	ref	ref	ref
	T2(≥0.908, <1.136; n=265)	1.22(0.89-1.68) 0.221	1.23(0.89-1.69) 0.208	1.17(0.85-1.61) 0.347	1.78(0.88-1.58) 0.272	1.19(0.89-1.59) 0.244	1.13(0.84-1.52) 0.410
	T3(≥1.136, <1.404; n=266)	1.21(0.88-1.67) 0.244	1.25(0.90-1.72) 0.181	1.18(0.85-1.64) 0.321	1.11(0.82-1.49) 0.499	1.15(0.85-1.54) 0.371	1.09(0.80-1.47) 0.599
	T4(≥1.404; n=266)	1.55(1.14-2.11) **0.006**	1.73(1.26-2.36) **<0.001**	1.60(1.15-2.23) **0.005**	1.38(1.04-1.83) **0.028**	1.53(1.15-2.05) **0.004**	1.41(1.04-1.92) **0.027**
	*P* for trend	**0.009**	**0.001**	**0.008**	0.050	**0.008**	**0.048**
	**Dichotomized groups** High (≥1.732) vs Low (<1.732)	1.96(1.48-2.61) **<0.001**	2.07(1.55-2.77) **<0.001**	1.79(1.32-2.44) **<0.001**	1.82(1.38-2.39) **<0.001**	1.92(1.45-2.53) **<0.001**	1.66(1.24-2.22) **<0.001**
TyG index	**Continuous**	1.14(1.00-1.31) 0.052	1.32(1.15-1.52) **<0.001**	1.20(1.02-1.40) **0.026**	1.10(0.97-1.25) 0.139	1.26(1.11-1.44) **<0.001**	1.14(0.98-1.32) 0.087
	Quartiles						
	T1(<1.093; n=266)	Ref	ref	ref	ref	ref	ref
	T2(≥1.093, <1.547; n=265)	1.19(0.87-1.63) 0.281	1.28(0.93-1.76) 0.128	1.30(0.94-1.80) 0.108	1.14(0.86-1.52) 0.366	1.22(0.92-1.64) 0.172	1.23(0.91-1.65) 0.174
	T3(≥1.547, <2.080; n=266)	1.26(0.92-1.72) 0.148	1.48(1.08-2.03) **0.015**	1.27(0.91-1.78) 0.160	1.14(0.85-1.53) 0.372	1.33(0.99-1.78) 0.059	1.13(0.83-1.54) 0.442
	T4(≥2.080; n=266)	1.28(0.94-1.75) 0.123	1.67(1.21-2.30) **0.002**	1.45(1.01-2.08) **0.044**	1.18(0.89-1.58) 0.257	1.51(1.12-2.04) **0.006**	1.29(0.92-1.80) 0.139
	*P* for trend	0.116	**0.001**	0.061	0.284	**0.006**	0.208
	**Dichotomized groups** High (≥2.582) vs Low (<2.582)	1.43(1.06-1.93) **0.019**	1.83(1.35-2.50) **<0.001**	1.54(1.10-2.14) **0.011**	1.33(1.00-1.77) 0.053	1.68(1.26-2.26) **<0.001**	1.44(1.05-1.97) **0.023**

HF, heart failure; SHR, stress hyperglycemia ratio; TyG, triglyceride-glucose.

Model 1: unadjusted.

Model 2: Adjusted for age, gender, race.

Model 3: Adjusted for age, gender, race, hypertension, diabetes, stroke, dyslipidemia, myocardial infarction, atrial fibrillation, chronic kidney disease, acute kidney injury, chronic obstructive pulmonary disease, respiratory failure.

Bold font indicates that the *P*-value is < 0.05 (representing statistical significance).

When analyzed as a continuous variable, TyG index was also associated with 180-day mortality risk in the adjusted models (Model 2: HR 1.32, 95% CI 1.15-1.52; Model 3: HR 1.20, 95% CI 1.02-1.40). Patients were then categorized into quartiles (T1: <1.093; T2: ≥1.093 to <1.547; T3: ≥1.547 to <2.080; T4: ≥2.080). In the highest quartile (T4), TyG index was positively associated with an increased risk of 180-day mortality in the multivariable models (Model 2: HR 1.67, 95% CI 1.21-2.30; Model 3: HR 1.45, 95% CI 1.01-2.08) (**Table 2**). Subsequently, trend tests and RCS analyses revealed that the association trend between TyG index and 180-day mortality risk differed across models. In model 2, a significant linear trend was observed (*P* for trend = 0.001) (**Table 2**), and no evidence of a nonlinear relationship was found in the RCS curve (*P* for nonlinear = 0.536) (**Figure 2**). In model 3, the linear trend was no longer significant (*P* for trend = 0.061) (**Table 2**), but the RCS analysis still visually tends to show a linear trend (**Figure 2**). Next, HF patients were divided into two groups based on the optimal cutoff value of TyG index: high (≥2.582) vs low (<2.582). It was found that the high group had an increased risk of mortality at 180 days (model1: HR 1.43, 95% CI 1.06-1.93; model2: HR 1.83, 95% CI 1.35-2.50; model3: HR 1.54, 95% CI 1.10-2.14) (**Table 2**).

### 3.3 Cox regression and RCS analyses of SHR and TyG index with 1-year, 90-day, 30-day mortality risk

Analyses of secondary outcomes revealed that SHR, as a continuous variable, was significantly associated with an increased risk of mortality at 365 days (Model 1: HR 1.26, 95% CI 1.06-1.49; Model 2: HR 1.35, 95% CI 1.13-1.60; Model 3: HR 1.26, 95% CI 1.04-1.52) (**Table 2**), 90 days (Model 1: HR 1.46, 95% CI 1.23-1.72; Model 2: HR 1.55, 95% CI 1.31-1.85; Model 3: HR 1.47, 95% CI 1.22-1.79) (**Supplementary Table S1**), and 30 days (Model 1: HR 1.57, 95% CI 1.32-1.88; Model 2: HR 1.64, 95% CI 1.36-1.98; Model 3: HR 1.53, 95% CI 1.24-1.89) (**Supplementary Table S2**). Similarly, patients in the highest SHR quartile (T4) exhibited significantly higher mortality risks at all these time points (all *P* < 0.05) (**Table 2**; **Supplementary Tables S1**, **S2**). Trend tests and RCS analyses consistently demonstrated significant linear relationships between SHR and mortality risk at 365, 90, and 30 days across the adjusted models (all *P* for trend < 0.05; all *P* for nonlinear > 0.05) (**Table 2**; **Supplementary Tables S1**, **S2**; **Figure 2**; **Supplementary Figures S1**, **S2**). Furthermore, the high group was consistently associated with a significantly increased risk of mortality at 365 days, 90 days, and 30 days (all *P* < 0.001) (**Table 2**; **Supplementary Table S3**).

When analyzed as a continuous variable, TyG index was associated with 1-year mortality in model 2, which adjusted for age, gender, and race (HR 1.26, 95% CI 1.11-1.44), but not in model 1 (unadjusted for covariates) or the model 3 (further adjusted for comorbidities) (*P*>0.05). A similar result was observed for the highest quartile (T4) (**Table 2**). Trend analysis and RCS curves indicated a significant positive linear trend for 1-year mortality in Model 2 (*P* for trend = 0.006; *P* for nonlinear = 0.849) (**Table 2**, **Figure 2**). In Model 3, this trend was attenuated and became non-significant (*P* for trend = 0.208), although no evidence of nonlinearity was observed (*P* for nonlinear = 0.804) (**Table 2**, **Figure 2**). Furthermore, in both model 2 and model 3, HF patients in the high group had a significantly increased 1-year mortality risk (all *P* < 0.001) (**Table 2**). For shorter-term outcomes, an elevated TyG index was consistently associated with increased 90-day and 30-day mortality across all models (all *P* < 0.05). Simultaneously, the highest TyG index quartile (T4) was significantly associated with increased 90-day mortality in adjusted models and with 30-day mortality across all models (**Supplementary Tables S1**, **S2**). Both trend tests and RCS analyses confirmed significant linear association between TyG index and the risks of 90-day and 30-day mortality (all *P* for trend < 0.05; all *P* for nonlinear > 0.05) (**Supplementary Tables S1**, **S2**; **Supplementary Figures S1**, **S2**). Finally, the high group demonstrated significantly increased risks for both 90-day and 30-day mortality in all models (all *P* < 0.001) (**Supplementary Table S3**).

### 3.4 K-M curves analyses for SHR, TyG index, and mortality risk

**Figure 3:**
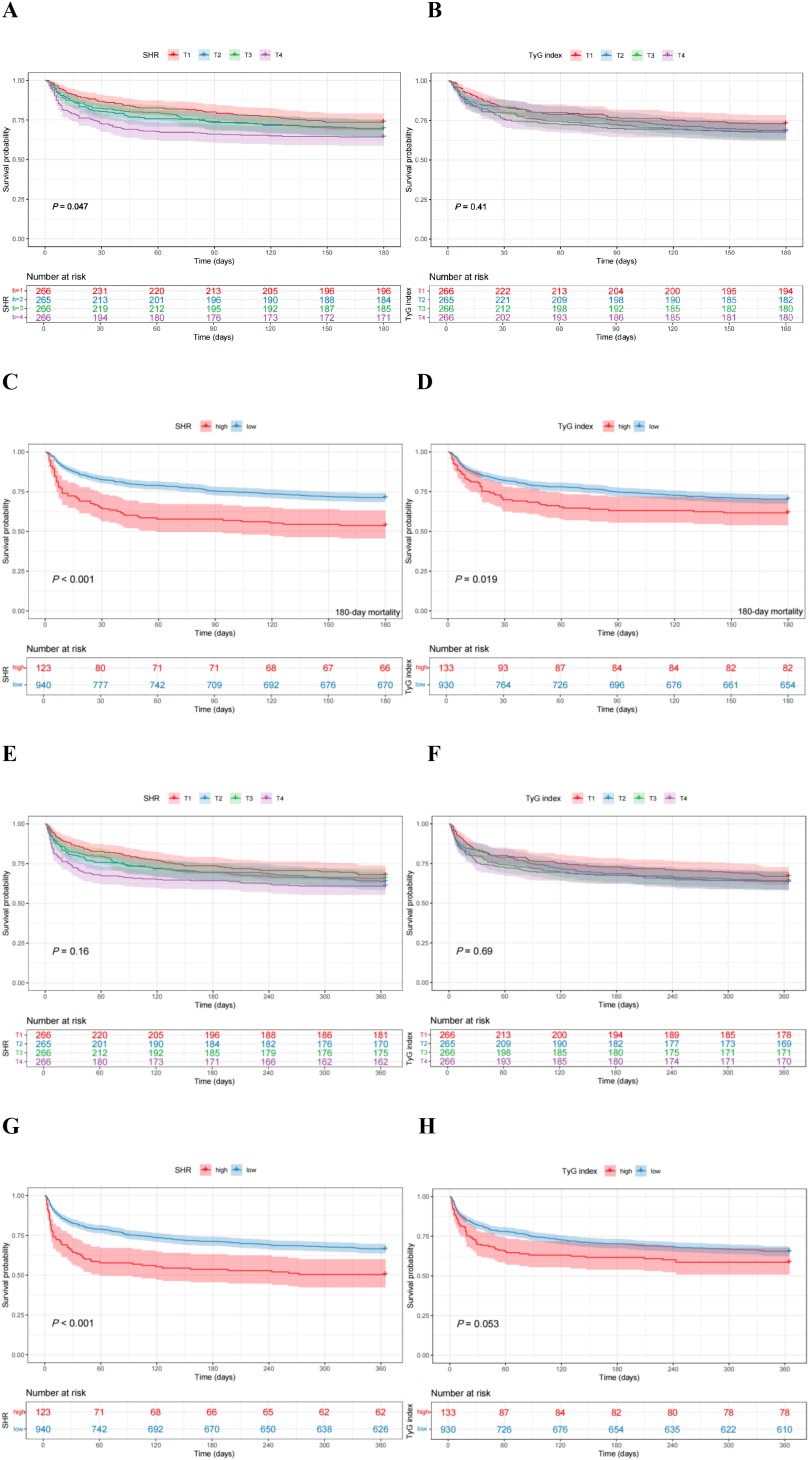
K-M curves of SHR and TyG index with 180-day and 1-year mortality [**A–D**, 180-day; **E–H**, 1-year. **(A, B, E, F)**, stratified by quartiles; **(C, D, G, H)**, dichotomized by optimal cutoffs].

When SHR was analyzed as a quartile variable, it showed a significant association with mortality at 180, 90, and 30 days, demonstrating a gradient of decreasing survival probability across higher quartiles (all *P* < 0.05) (**Figure 3**, **Supplementary Figure S3**). However, no significant difference in 1-year mortality was observed among the four groups (*P* > 0.05) (**Figure 3**). When patients were dichotomized into high and low SHR groups based on the optimal cutoff value, the high SHR group had a significantly higher risk of both the primary outcome (180-day mortality) and all secondary outcomes (1-year, 90-day, and 30-day mortality) (all *P* < 0.001) (**Figure 3**; **Supplementary Figure S4**).

Unlike SHR, no significant differences in the risks of either primary or secondary outcomes were observed when TyG index was treated as a quartile variable (all *P* > 0.05) (**Figure 3**; **Supplementary Figure S3**). However, after being stratified into high and low groups based on the optimal cutoff, the high TyG group demonstrated significantly higher 180-day, 90-day, and 30-day mortality risks (all *P* < 0.05), but not 1-year mortality (*P* > 0.05) (**Figure 3**; **Supplementary Figure S4**).

### 3.5 Comparison of ROC curves for SHR and TyG Index

**Figure 4:**
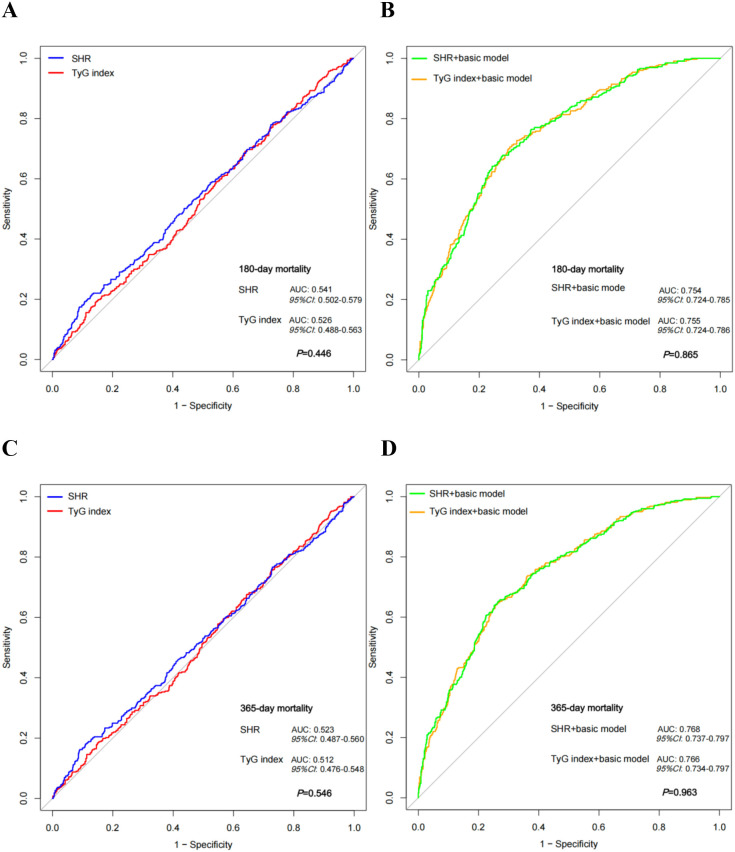
ROC curves of SHR and TyG index for predicting mortality [**A, B**, 180-day; **C, D**, 365-day. **(A, C)**, SHR vs TyG index; **(B, D)**, SHR+basic model vs TyG index+basic model].

ROC curve analysis demonstrated no significant difference in the predictive performance for 180-day mortality between SHR and TyG index in HF patients (AUC_SHR_ vs AUC_TyG index_: 0.541 vs 0.526, *P* = 0.446) (**Figure 4**). Similarly, no statistically significant differences were observed for the prediction of 1-year, 90-day, or 30-day mortality (all *P* > 0.05) (**Figure 4**; **Supplementary Figure S5**). The basic model was constructed using age, gender, race, hypertension, diabetes, stroke, dyslipidemia, MI, AF, CKD, AKI, COPD and RF. When SHR and TyG index were individually added to this basic model, both new composite models demonstrated significantly improved predictive ability compared to either index alone. However, there was no significant difference in predictive performance between the two composite models for 180-day (AUC_SHR+basic model_ vs AUC_TyG index+basic model_: 0.754 vs 0.755, *P* = 0.865), 365-day, 90-day, or 30-day mortality risk (all *P*>0.05) (**Figure 4**; **Supplementary Figure S5**).

### 3.6 Subgroup analysis

**Figure 5:**
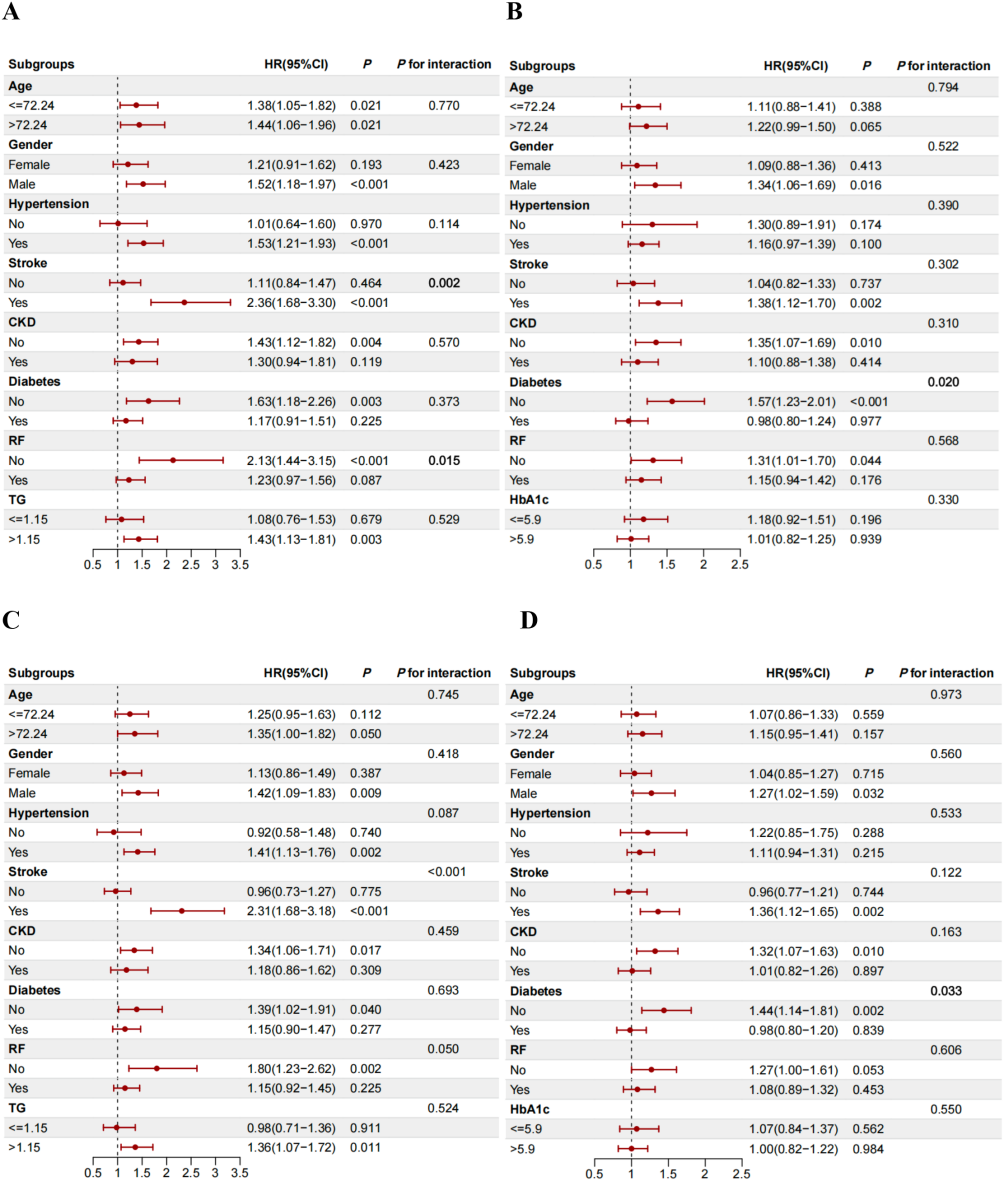
Associations of SHR and TyG index with 180-day and 1-year mortality by subgroups. **(A, B)** 180-day; **(C, D)**, 1-year. **(A, C)**, SHR; **(B, D)**, TyG index.

Subgroup analysis identified significant interactions for stroke (*P* for interaction = 0.002) and respiratory failure (*P* for interaction = 0.015) on the association between SHR and 180-day mortality. Notably, patients with a history of stroke (HR 2.36, 95% CI 1.68-3.30) and those without respiratory failure (HR 2.13, 95% CI 1.44-3.15) exhibited a significantly elevated risk of 180-day mortality (**Figure 5**). Similar patterns of association were observed for 365-day, 90-day, and 30-day mortality (**Figure 5**; **Supplementary Figures S6**, **S7**).

For TyG index, the association with 180-day mortality was significantly modified by diabetes status (*P* for interaction = 0.015). Non-diabetic patients exhibited a significantly higher risk (HR 1.57, 95% CI 1.23-2.01) (**Figure 5**). This pattern was consistent for mortality at 365, 90, and 30 days (**Figure 5**; **Supplementary Figures S6**, **S7**). Furthermore, the association between TyG index and 30-day mortality also demonstrated a significant interaction in the RF subgroup (*P* for interaction = 0.039), with a higher risk observed in patients without RF (**Supplementary Figure S7**).

## 4 Discussion

### 4.1 Study findings

This study is the first to compare SHR and TyG index in critically ill HF patients in terms of their associations and predictive performance with mortality risk. The principal findings are summarized: (1) SHR is significantly associated with the 180-day, 1-year, 90-day, and 30-day mortality risks in critically ill HF patients, whereas TyG index is only associated with the 180-day, 90-day, and 30-day mortality risks, and not with the 1-year mortality risk. (2) The aforementioned associations are primarily linear. (3) Compared to their respective lowest quartiles, the highest quartile of SHR was associated with significantly increased mortality risks across all time points, however, Kaplan-Meier curves indicated no difference in 1-year survival among the four SHR quartiles; the highest quartile of TyG index had increased mortality risk at 180-day, 90-day, and 30-day, yet K-M curves showed no significant survival differences among quartiles at any time point. (4) When SHR and TyG index were used as dichotomous variables, mortality risks in the high groups were significantly greater at all time points, with the sole exception that the 1-year mortality of the high TyG group was not increased compared to the low group in the unadjusted model, which was consistent with the Kaplan-Meier analysis. (5) The predictive performance of SHR and TyG index for the primary and secondary outcomes was comparable. Furthermore, the enhanced models created by individually adding SHR or TyG index to the basic model also showed no statistically significant difference in discriminatory power. (6) The association of SHR with mortality across all time points was strengthened in subgroups of patients with stroke and without RF. Meanwhile, TyG index exhibited stronger association with mortality in non-diabetic patients, additionally, its association with 30-day mortality was particularly enhanced in patients without RF.

### 4.2 Rationale, necessity, and interpretation of key findings from comparing SHR and TyG index

Multiple studies have demonstrated that glucose metabolic disorders, which are common in critically ill patients, are strongly associated with adverse outcomes, including increased mortality (6, 20). In recent years, SHR and TyG index have garnered significant attention as indicators of glucose metabolic status (11, 12, 17), not only due to their simplicity, cost-effectiveness, and accessibility but also because of their established effectiveness in assessing the risk of adverse outcomes across various diseases. By integrating background blood glucose, SHR more accurately reflects the acute glycemic state in critically ill patients than absolute blood glucose (21) and has been extensively associated with poor outcomes in cerebrovascular, infectious diseases, acute pancreatitis, and malignancies (12). Similarly, TyG index as a reliable marker of insulin resistance is closely linked to systemic glucose and lipid metabolism (22). Higher TyG index values are strongly correlated with an increased risk of adverse events, including mortality, in various cardiovascular diseases such as AF (23), ischemic stroke (24), acute MI (16), and acute decompensated heart failure (25). Yet, the core question of whether SHR or TyG index serves as a better predictor of adverse prognosis has not been definitively answered by prior research. Our study identified the SHR as a more applicable and robust metabolic biomarker for 1-year mortality, whereas the TyG index demonstrated no significant association. The finding regarding TyG index is consistent with the conclusions reached by Jing Xiao et al. (26). Grounded in clinical experience and their respective formulas, the superiority of SHR over TyG index can be attributed to the following two aspects: (1) The SHR, by quantifying the disparity between acute and chronic glucose levels (effectively transforming absolute glucose into a relative metric), more accurately reflects the body’s stress state. In addition, the intensity of stress reflects the body’s intrinsic regulatory capacity and tolerance. This means that the SHR is a superior indicator of the severity of critical illness and overall vulnerability than the TyG index. (2) TG levels are more susceptible than HbA1c to influences from parenteral nutrition, medications, and hepatic or renal dysfunction, exhibiting greater short-term variability that may attenuate the association between the TyG index and 1-year outcomes.

### 4.3 Mechanisms of the association between SHR, TyG index, and mortality

Analysis of the association trends of both SHR and TyG index with primary and secondary outcomes revealed a predominantly linear relationship, which reflects that elevated blood glucose levels confer a poorer prognosis. The underlying mechanisms are multifactorial. Firstly, elevated blood glucose promotes cytokine production, oxidative stress, inflammatory responses, and endothelial dysfunction (12, 27, 28), leading to cardiomyocyte injury and aggravated HF. The critical HF state, in turn, triggers heightened sympathetic activity, wherein catecholamines inhibit insulin secretion and promote glycogenolysis, resulting in further hyperglycemia (6, 28) and thereby establishing a vicious cycle. Secondly, hyperglycemia promotes procoagulant responses (29) and reduces fibrinolytic activity (30), while also enhancing platelet activation and aggregation through multiple mechanisms (31). Moreover, in critically ill HF patients, prolonged bed rest further contributes to thrombus formation, thereby exacerbating circulatory dysfunction. Thirdly, hyperglycemia increases the risk of nosocomial infection during hospitalization (32). It impairs the function of various immune cells (33), creates a microenvironment conducive to bacterial proliferation and enhanced virulence, and facilitates the development of pathogen drug resistance (34). These alterations collectively lead to persistent and refractory infections (35). Lastly, hyperglycemia promotes atherosclerosis, a significant risk factor for coronary artery disease (CAD). Given that CAD is the primary etiology of HF (36), a vicious cycle is established between myocardial ischemia and HF, thereby driving progressive HF exacerbation. Concurrently, renal artery atherosclerosis can lead to renal artery stenosis and hypoperfusion, resulting in renal dysfunction, thus culminating in the vicious cycle of cardiorenal syndrome (37).

### 4.4 Analysis of subgroup results

Elevated SHR significantly increased mortality risk across all time points in the stroke subgroup. This may be attributable to their pre-existing neurological decline and pathophysiological changes, including endothelial dysfunction and atherosclerosis (38, 39). While the onset of HF represents a significant additional insult to the organism, in this vulnerable state, elevated SHR exacerbates injury to both cerebral tissue and the vasculature, thereby creating a vicious cycle that significantly increases mortality. Simultaneously, in patients without RF, the occurrence of outcome events is more likely attributable to non-respiratory diseases, such as progressively worsening HF, advancement to cardiogenic shock, or malignant ventricular arrhythmias. Glucose metabolic dysregulation acts as a key pathogenic driver of this process (40–42). TyG index demonstrated a significantly stronger association with increased mortality in non-diabetic individuals, whereas no significant correlation was observed in the diabetic subgroup. This discrepancy may be attributed to chronic hyperglycemia in diabetes, which can induce a state of glucotoxic tolerance (28). Consequently, even at comparable absolute glucose levels, the relative glycemic excursion is smaller in diabetic patients, thereby attenuating the associated pathophysiological impact and the prognostic power of TyG index. Previous studies have also indicated that elevated blood glucose exerts more severe effects in non-diabetic individuals, signified by a markedly increased risk of cardiovascular and cerebrovascular mortality (43). Consistently, strict glycemic control in ICU patients significantly reduced mortality in those without diabetes, but conferred no survival benefit to diabetic patients (44). These findings collectively indicate that both hyperglycemia and its fluctuations pose a greater pathophysiological threat to non-diabetic individuals.

### 4.5 Clinical significance

In clinical practice, prioritizing the acquisition of SHR values is not only efficient, cost-effective, and resource-saving, but it is also crucial for delaying disease progression and reducing mortality in critically ill HF patients through early risk stratification and timely intervention. From a broader perspective, the results of this study may also provide valuable insights for the long-term (5, 10, or even 20 years) prognostic biomarker research and selection in HF.

### 4.6 Limitations

Despite the numerous innovative findings and clinically significant conclusions of our study, several limitations must be acknowledged: (1) As a single-center, retrospective cohort study, this research is inherently prone to selection and information biases. Therefore, the findings require validation in future multicenter, prospective cohort studies to enhance their clinical applicability. Meanwhile, we note that the CHA_2_DS_2_-VASc score, commonly used for thromboembolic risk assessment in patients with AF, has also demonstrated significant predictive value for mortality in HF patients (45). And the positive association remains significant even after adjustment for AF (46). Therefore, in future multicenter and prospective cohort studies, we plan to incorporate the CHA_2_DS_2_-VASc score into our analysis to maximize the sensitivity and specificity of HF prognosis prediction. (2) Although we have adjusted for known confounders and conducted subgroup analyses, there may still be potential and unknown confounders, such as education level, socioeconomic status, and psychological factors, that could interfere with the results. (3) The lack of dynamic monitoring of SHR and TyG index in this study precludes the capture of their potential significant fluctuations. These variations may affect the stability of the assessed risk for outcome events. Therefore, future research is warranted to develop trajectory models for these metrics, which would provide a more robust theoretical foundation for prognosis assessment and personalized management. (4) Given the observational design of this study, causal inferences are limited. To address this, future research could adopt causal analytic frameworks like structural equation modeling (e.g., Liu et al. (47)) or investigate genetic causality via Mendelian randomization, as demonstrated in studies including Li et al. (48).

## 5 Conclusion

SHR demonstrated significant associations with 30-, 90-, 180-, and 365-day mortality in critically ill HF patients. In contrast, the TyG index was associated only with short-term mortality (30, 90, and 180 days) but not with the 365-day outcome. However, the predictive performance of the two indicators for short-term mortality did not differ significantly. Overall, SHR and TyG index can serve as potential risk assessment tools for short-term (180-day, 90-day, 30-day) mortality risk in HF patients. However, for the assessment of 1-year mortality risk, SHR is the more applicable and robust metabolic indicator, as it maintains a significant association where the TyG index does not.

## Data Availability

Publicly available datasets were analyzed in this study. This data can be found here: https://physionet.org/content/mimiciv/3.1/.
