# Supplementary material for: Head-to-head comparison of stress hyperglycemia ratio versus triglyceride-glucose index for predicting mortality in heart failure: a retrospective cohort study
Source: Front Endocrinol (Lausanne). 2026 Feb 12;17:1782922. doi: 10.3389/fendo.2026.1782922 (PMC12935658; doi:10.3389/fendo.2026.1782922)
Supplement: Supplementary file 1 [file DataSheet1.zip › supplementary material/Supplementary material 2.docx]

**Supplementary Material 2**

**Table S1** Associations of SHR and TyG index with 90-day mortality in HF patients.

**Table S2** Associations of SHR and TyG index with 30-day mortality in HF patients.

**Table S3** Associations of SHR and TyG index with 90-day and 30-day mortality in HF patients, dichotomized using optimal cutoffs.

**Fig.S1** After adjustment for age, sex, and race, the RCS curves of SHR and TyG index with 90-day and 30-day mortality in HF patients.

**Fig.S2** After adjustment for age, gender, race, hypertension, diabetes, stroke, dyslipidemia, myocardial infarction, atrial fibrillation, chronic kidney disease, acute kidney injury, chronic obstructive pulmonary disease, respiratory failure, the RCS curves of SHR and TyG index with 90-day and 30-day mortality in HF patients.

**Fig.S3** K-M curves of SHR and TyG index with 90-day and 30-day mortality in HF patients, stratified by quartiles.

**Fig.S4** K-M curves of SHR and TyG index with 90-day and 30-day mortality in HF patients, dichotomized by optimal cutoffs.

**Fig.S5** ROC curves of TyG index and SHR for predicting 90-day and 30-day mortality in HF patients.

**Fig.S6** Associations of SHR and TyG index with 90-day mortality in HF patients by subgroups.

**Fig.S7** Associations of SHR and TyG index with 30-day mortality in HF patients by subgroups.

**Table S1**

Associations of SHR and TyG index with 90-day mortality in HF patients.

| **Variables** | **Groups** | **Model 1** | **Model 2** | **Model 3** |
| --- | --- | --- | --- | --- |
|  |  | **HR (95% CI) *P*-Value** | **HR (95% CI) *P*-Value** | **HR (95% CI) *P*-Value** |
| SHR | Continuous | 1.46(1.23-1.72) **<0.001** | 1.55(1.31-1.85) **<0.001** | 1.47(1.22-1.79) **<0.001** |
|  | T1(<0.908; n=266) | ref | ref | ref |
|  | T2(≥0.908, <1.136; n=265) | 1.37(0.96-1.96) 0.084 | 1.37(0.95-1.96) 0.089 | 1.28(0.89-1.84) 0.183 |
|  | T3(≥1.136, <1.404; n=266) | 1.40(0.98-1.99) 0.066 | 1.42(0.99-2.03) 0.056 | 1.36(0.94-1.96) 0.099 |
|  | T4(≥1.404; n=266) | 1.93(1.37-2.71) **<0.001** | 2.12(1.51-2.99) **<0.001** | 2.00(1.40-2.88) **<0.001** |
|  | *P* for trend | **<0.001** | **<0.001** | **0.029** |
| TyG index | Continuous | 1.20(1.04-1.38) **0.013** | 1.38(1.19-1.60) **<0.001** | 1.24(1.05-1.46) **0.012** |
|  | T1(<1.093; n=266) | ref | ref | ref |
|  | T2(≥1.093, <1.547; n=265) | 1.11(0.79-1.57) 0.546 | 1.19(0.84-1.69) 0.323 | 1.22(0.86-1.73) 0.271 |
|  | T3(≥1.547, <2.080; n=266) | 1.26(0.90-1.76) 0.188 | 1.47(1.04-2.07) **0.027** | 1.25(0.87-1.80) 0.221 |
|  | T4(≥2.080; n=266) | 1.38(0.99-1.92) 0.057 | 1.79(1.27-2.51) **<0.001** | 1.53(1.04-2.25) **0.030** |
|  | *P* for trend | **0.042** | **<0.001** | **0.037** |

HF: heart failure; SHR: stress hyperglycemia ratio; TyG: triglyceride-glucose

Model 1: unadjusted

Model 2: Adjusted for age, gender, race

Model 3: Adjusted for age, gender, race, hypertension, diabetes, stroke, dyslipidemia, myocardial infarction, atrial fibrillation, chronic kidney disease, acute kidney injury, chronic obstructive pulmonary disease, respiratory failure

**Table S2**

Associations of SHR and TyG index with 30-day mortality in HF patients.

| **Variables** | **Groups** | **Model 1** | **Model 2** | **Model 3** |
| --- | --- | --- | --- | --- |
|  |  | **HR (95% CI) *P*-Value** | **HR (95% CI) *P*-Value** | **HR (95% CI) *P*-Value** |
| SHR | Continuous | 1.57(1.32-1.88) **<0.001** | 1.64(1.36-1.98) **<0.001** | 1.53(1.24-1.89) **<0.001** |
|  | T1(<0.908; n=266) | ref | ref | ref |
|  | T2(≥0.908, <1.136; n=265) | 1.55(1.01-2.38) **0.045** | 1.52(0.99-2.34) 0.055 | 1.38(0.89-2.13) 0.146 |
|  | T3(≥1.136, <1.404; n=266) | 1.39(0.90-2.16) 0.138 | 1.40(0.90-2.17) 0.134 | 1.30(0.83-2.03) 0.25 |
|  | T4(≥1.404; n=266) | 2.31(1.54-3.46) **<0.001** | 2.47(1.64-3.70) **<0.001** | 2.22(1.45-3.40) **<0.001** |
|  | *P* for trend | **<0.001** | **<0.001** | **0.041** |
| TyG index | Continuous | 1.30(1.10-1.52) **0.002** | 1.47(1.24-1.73) **<0.001** | 1.31(1.08-1.59) **0.007** |
|  | T1(<1.093; n=266) | ref | ref | ref |
|  | T2(≥1.093, <1.547; n=265) | 1.04(0.68-1.57) 0.868 | 1.09(0.72-1.66) 0.681 | 1.11(0.73-1.70) 0.632 |
|  | T3(≥1.547, <2.080; n=266) | 1.29(0.86-1.91) 0.218 | 1.46(0.98-2.19) 0.065 | 1.21(0.79-1.85) 0.380 |
|  | T4(≥2.080; n=266) | 1.56(1.07-2.29) **0.022** | 1.96(1.32-2.90) **<0.001** | 1.61(1.04-2.51) **0.034** |
|  | *P* for trend | **0.011** | **<0.001** | **0.036** |

HF: heart failure; SHR: stress hyperglycemia ratio; TyG: triglyceride-glucose

Model 1: unadjusted

Model 2: Adjusted for age, gender, race

Model 3: Adjusted for age, gender, race, hypertension, diabetes, stroke, dyslipidemia, myocardial infarction, atrial fibrillation, chronic kidney disease, acute kidney injury, chronic obstructive pulmonary disease, respiratory failure

**Table S3**

Associations of SHR and TyG index with 90-day and 30-day mortality in HF patients, dichotomized using optimal cutoffs.

| outcome | group | SHR (≥1.732 **vs** <1.732) | TyG index (≥2.582 **vs** <2.582) |
| --- | --- | --- | --- |
|  |  | HR (95% CI) *P*-Value | HR (95% CI) *P*-Value |
| 90-day mortality | model1 | 2.09(1.54-2.83) **<0.001** | 1.59(1.18-2.13) **0.002** |
|  | model2 | 2.18(1.60-2.96) **<0.001** | 1.97(1.45-2.67) **<0.001** |
|  | model3 | 1.86(1.35-2.58) **<0.001** | 1.70(1.22-2.37) **0.002** |
| 30-day mortality | model1 | 2.48(1.78-3.46) **<0.001** | 1.83(1.33-2.52) **<0.001** |
|  | model2 | 2.50(1.78-3.50) **<0.001** | 2.22(1.60-3.08) **<0.001** |
|  | model3 | 2.05(1.44-2.94) **<0.001** | 1.88(1.30-2.71) **<0.001** |

HF: heart failure; SHR: stress hyperglycemia ratio; TyG: triglyceride-glucose

Model 1: Unadjusted

Model 2: Adjusted for age, gender, race

Model 3: Adjusted for age, gender, race, hypertension, diabetes, stroke, dyslipidemia, myocardial infarction, atrial fibrillation, chronic kidney disease, acute kidney injury, chronic obstructive pulmonary disease, respiratory failure

**A B**


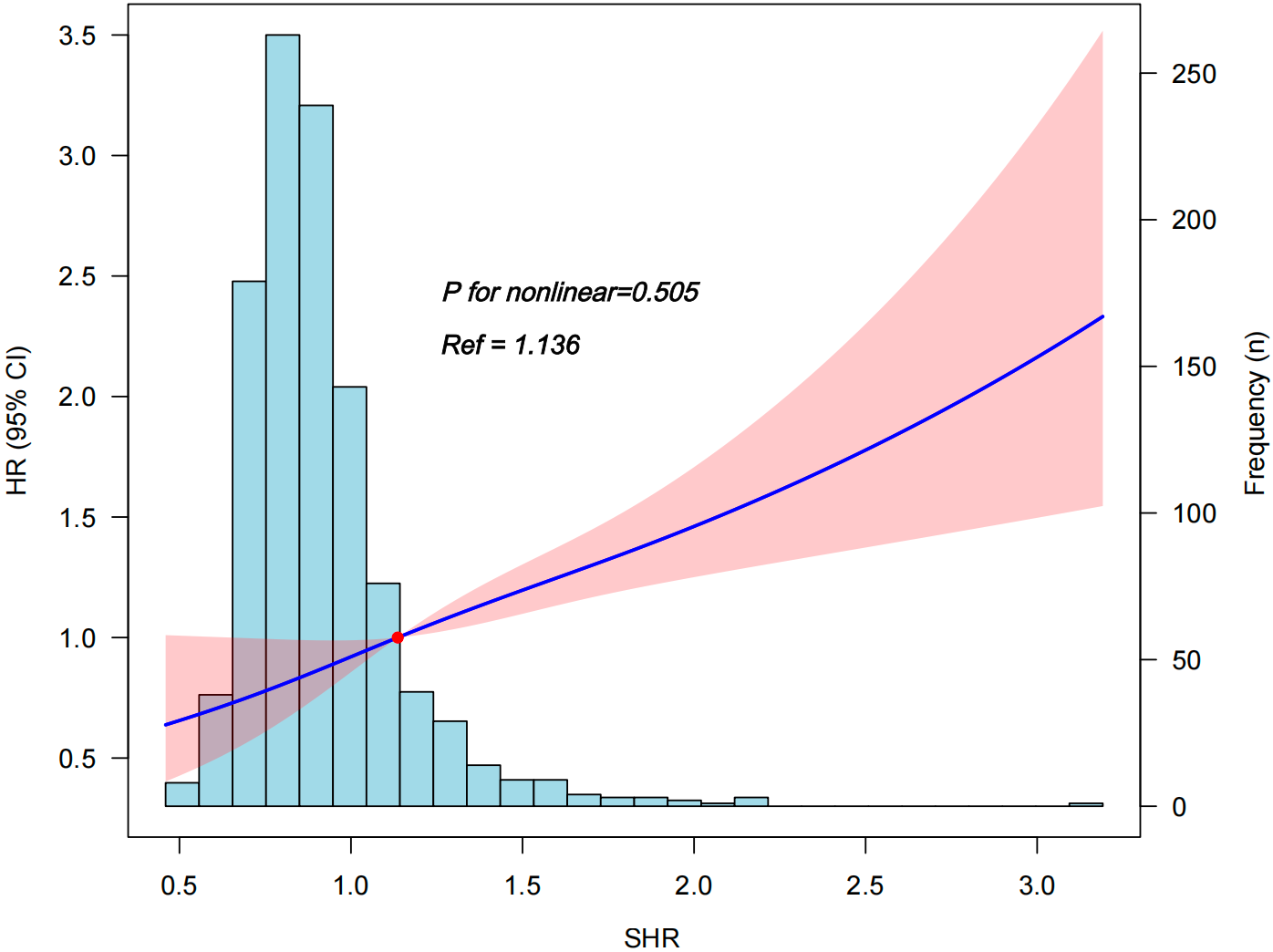

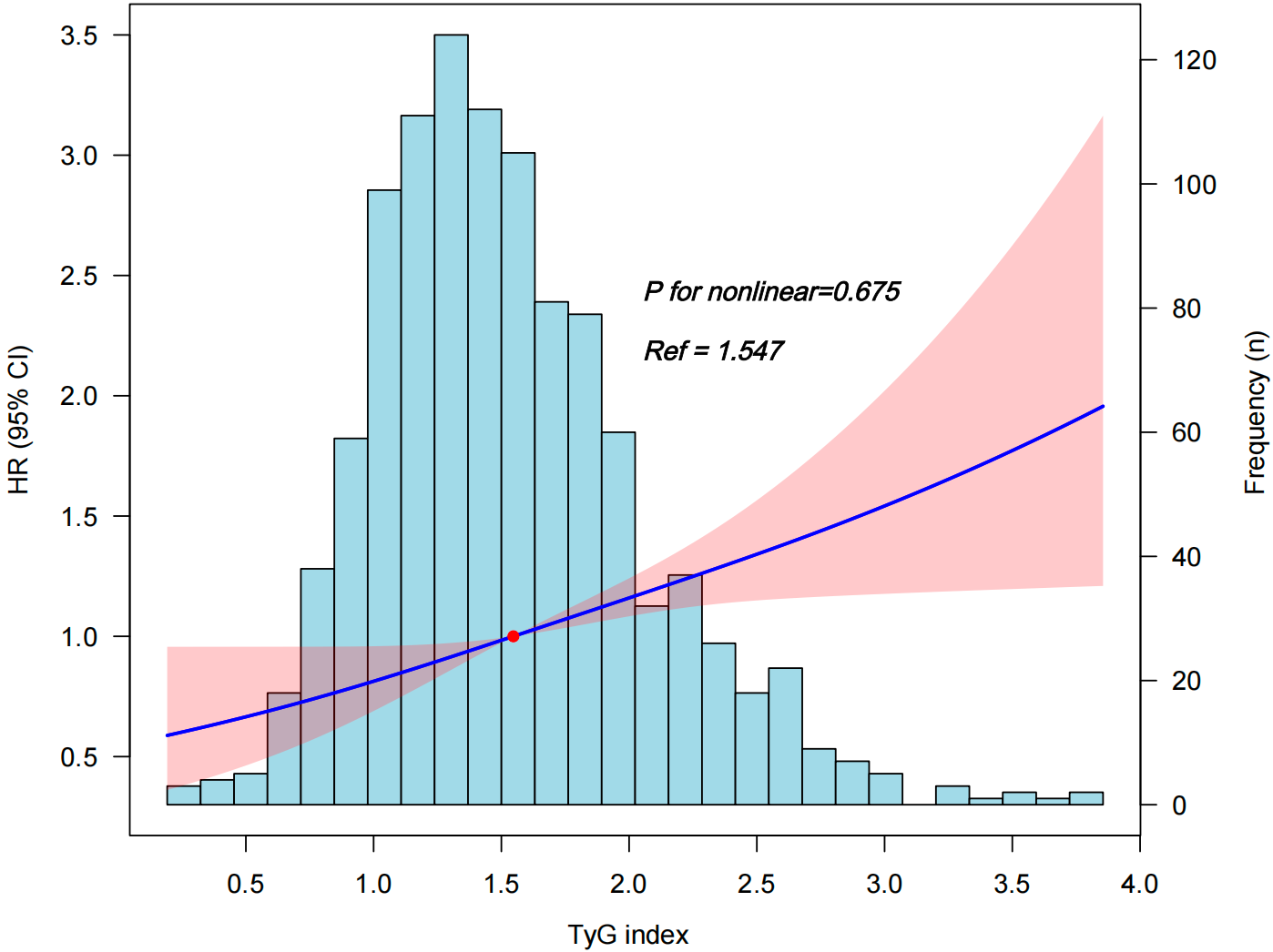


**C D**

**
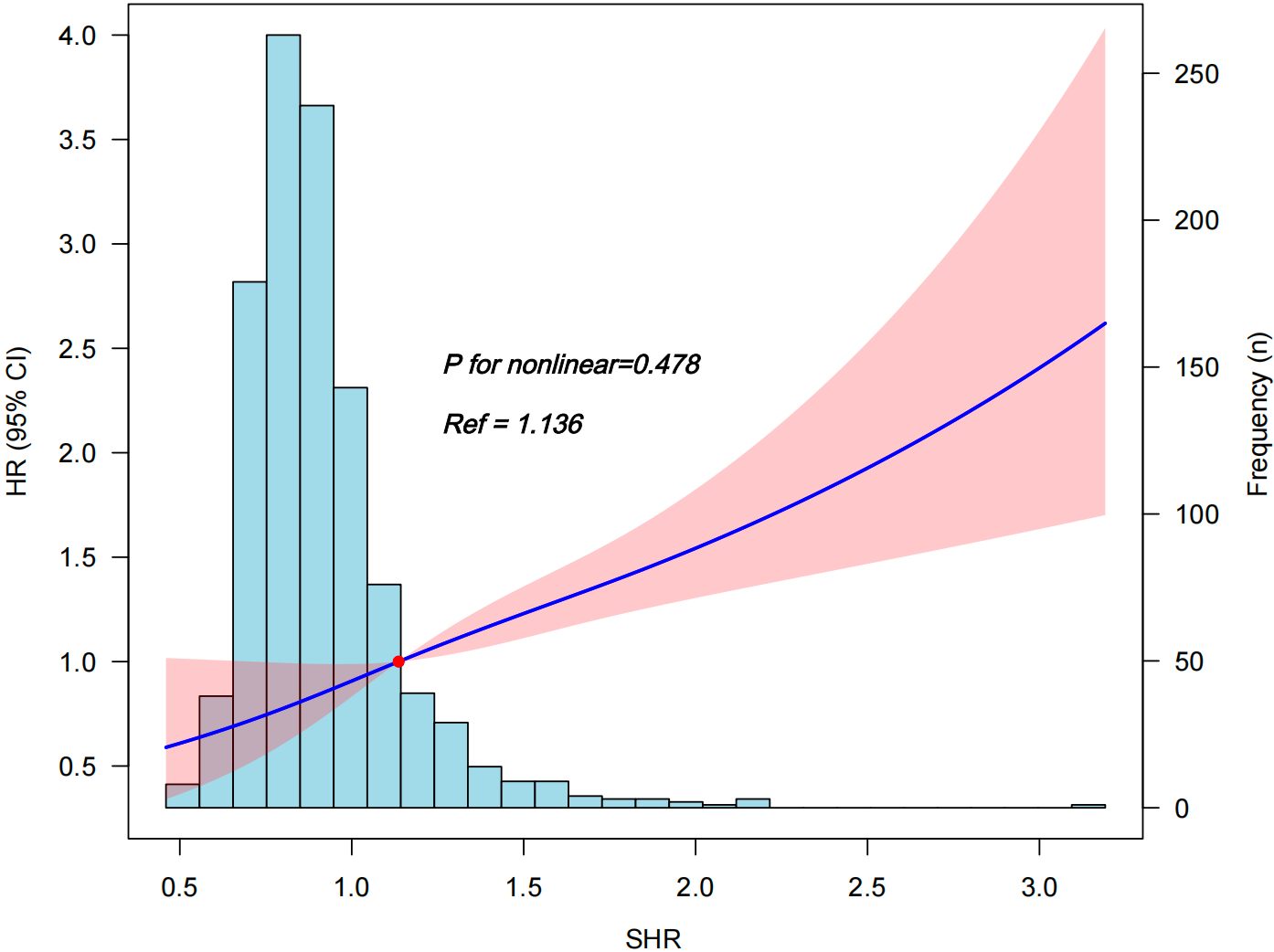

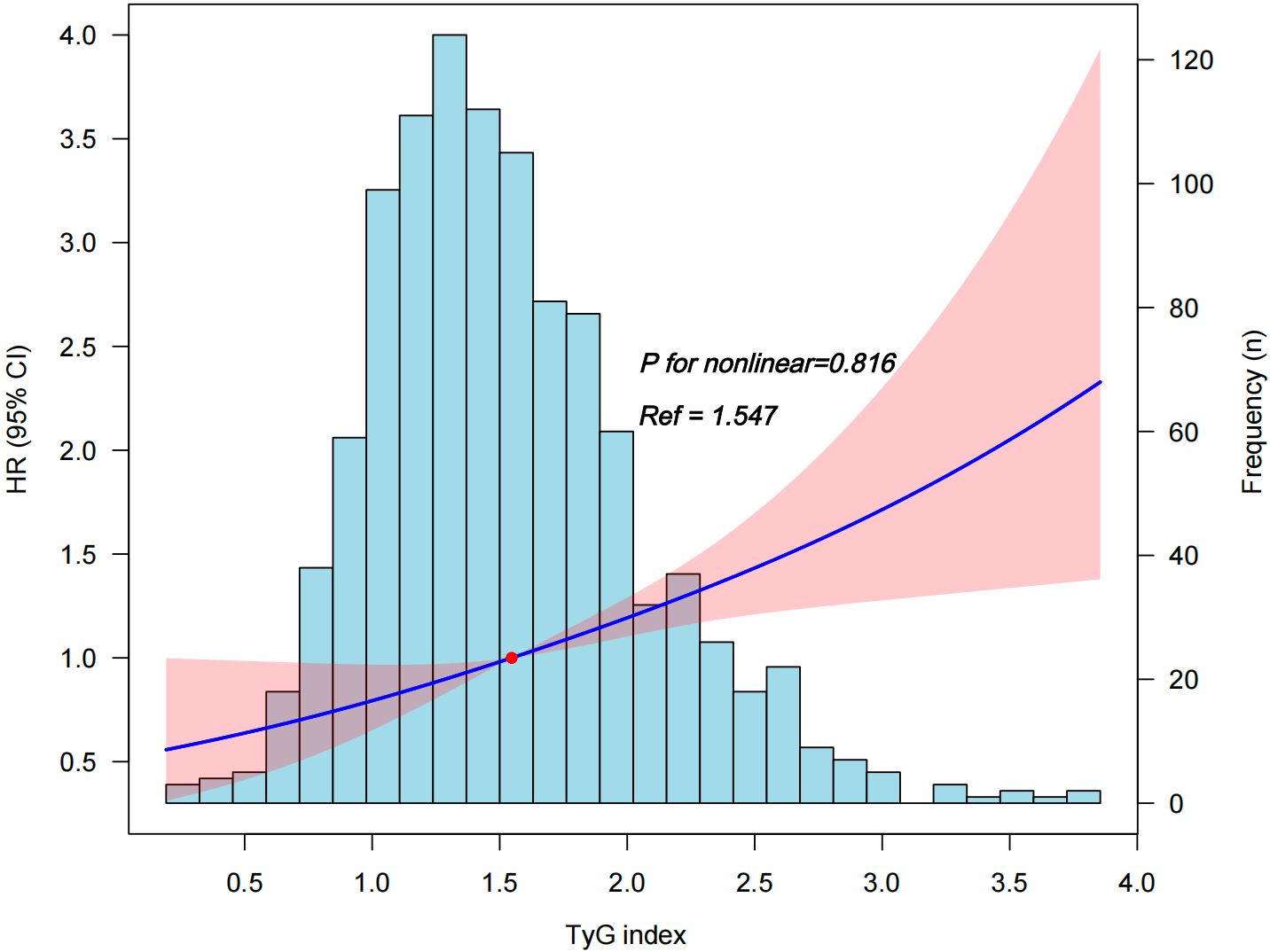
**

**Fig.S1** After adjustment for age, sex, and race, the RCS curves of SHR and TyG index with mortality in HF patients. **A** and **B**, 90-day; **C** and **D**, 30-day.

**A B**


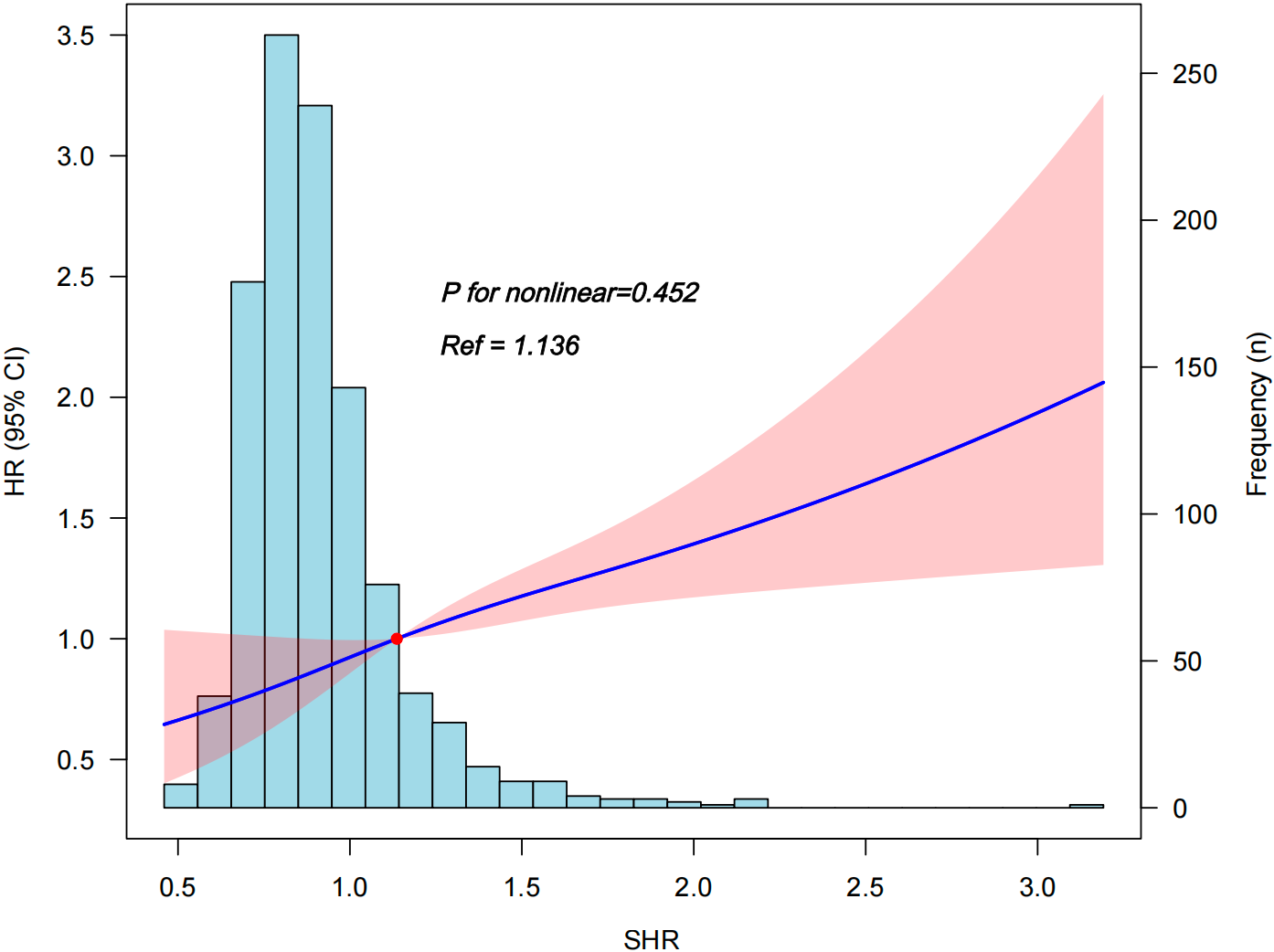

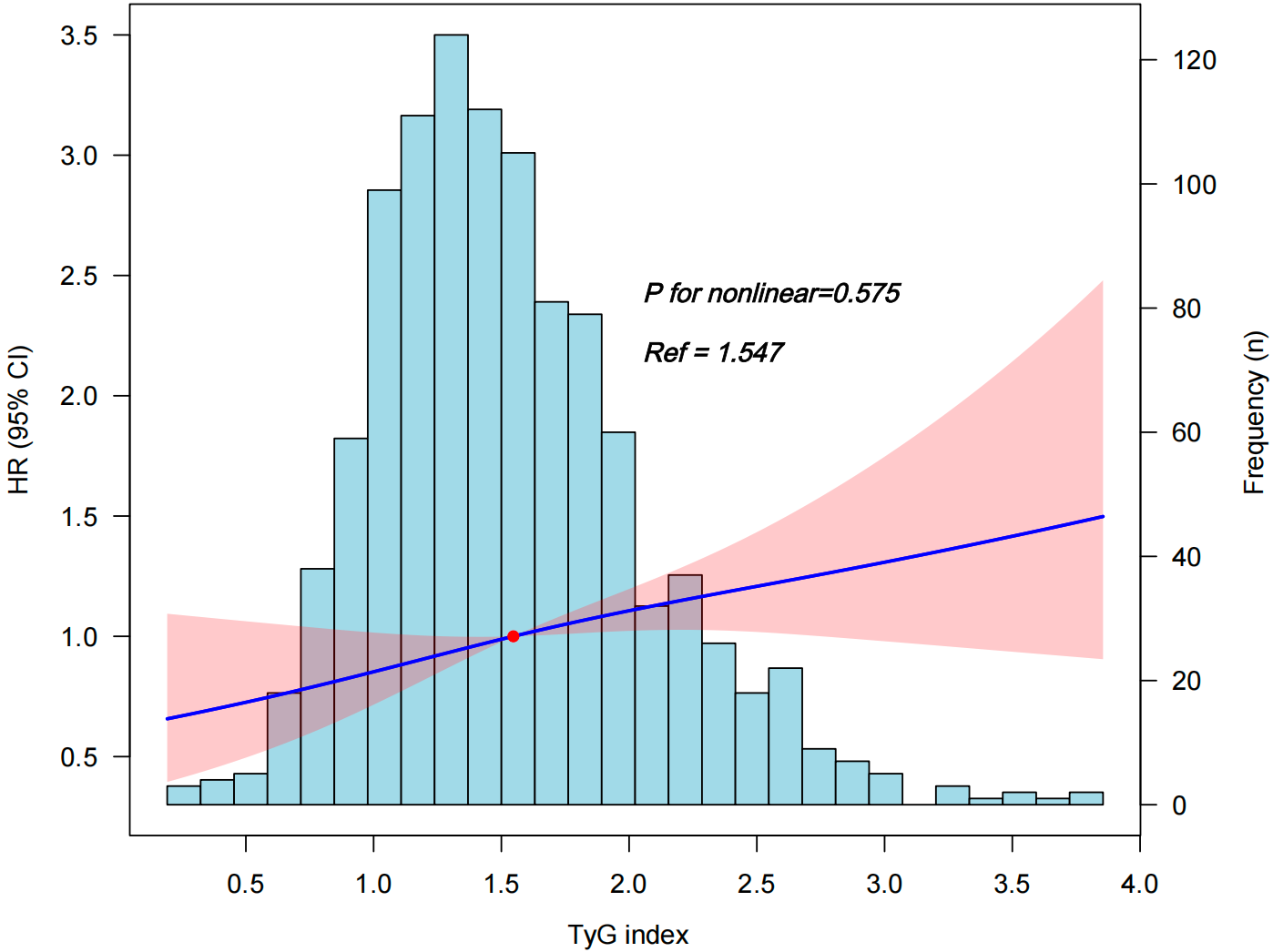


**C D**

**
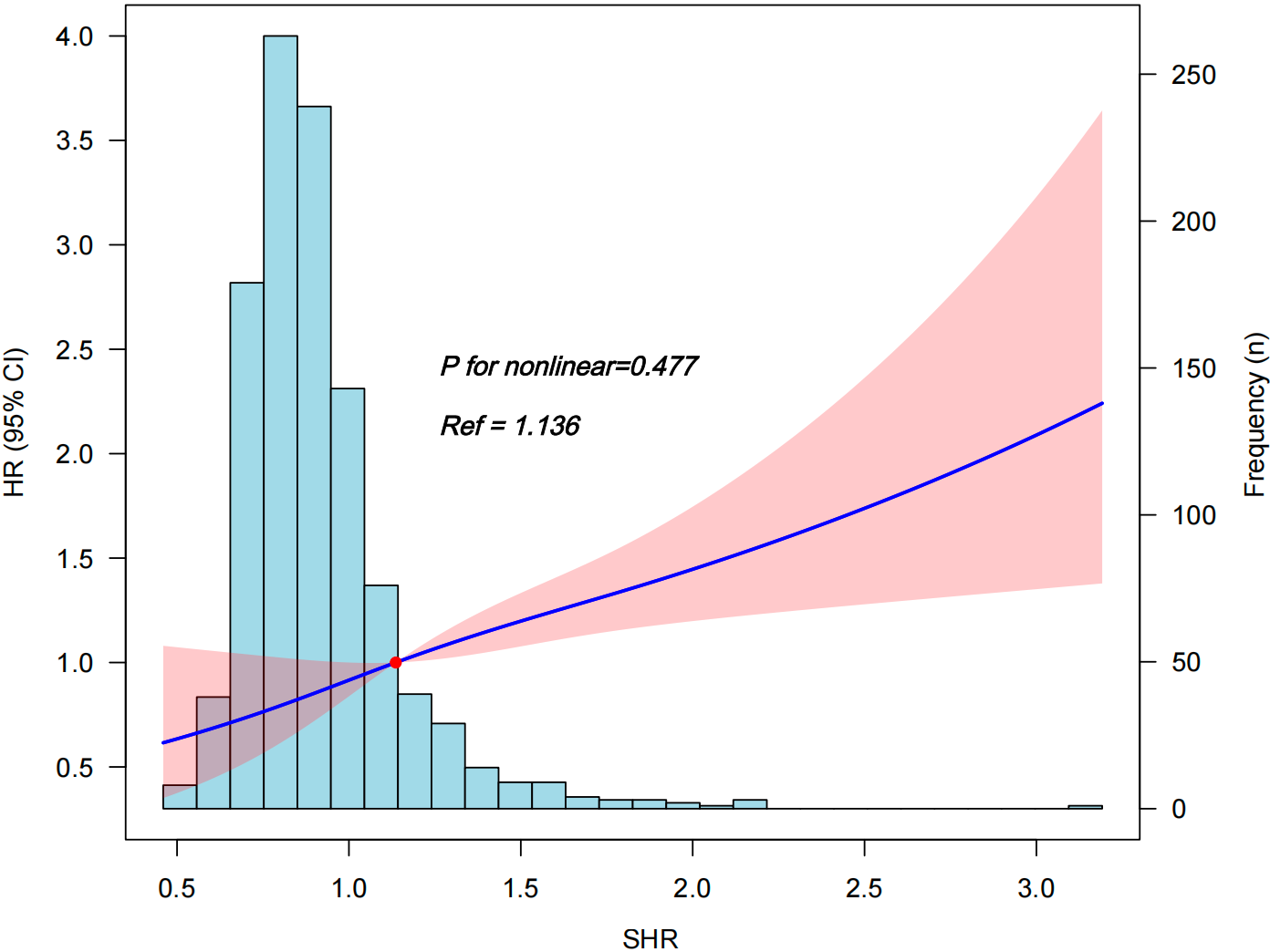

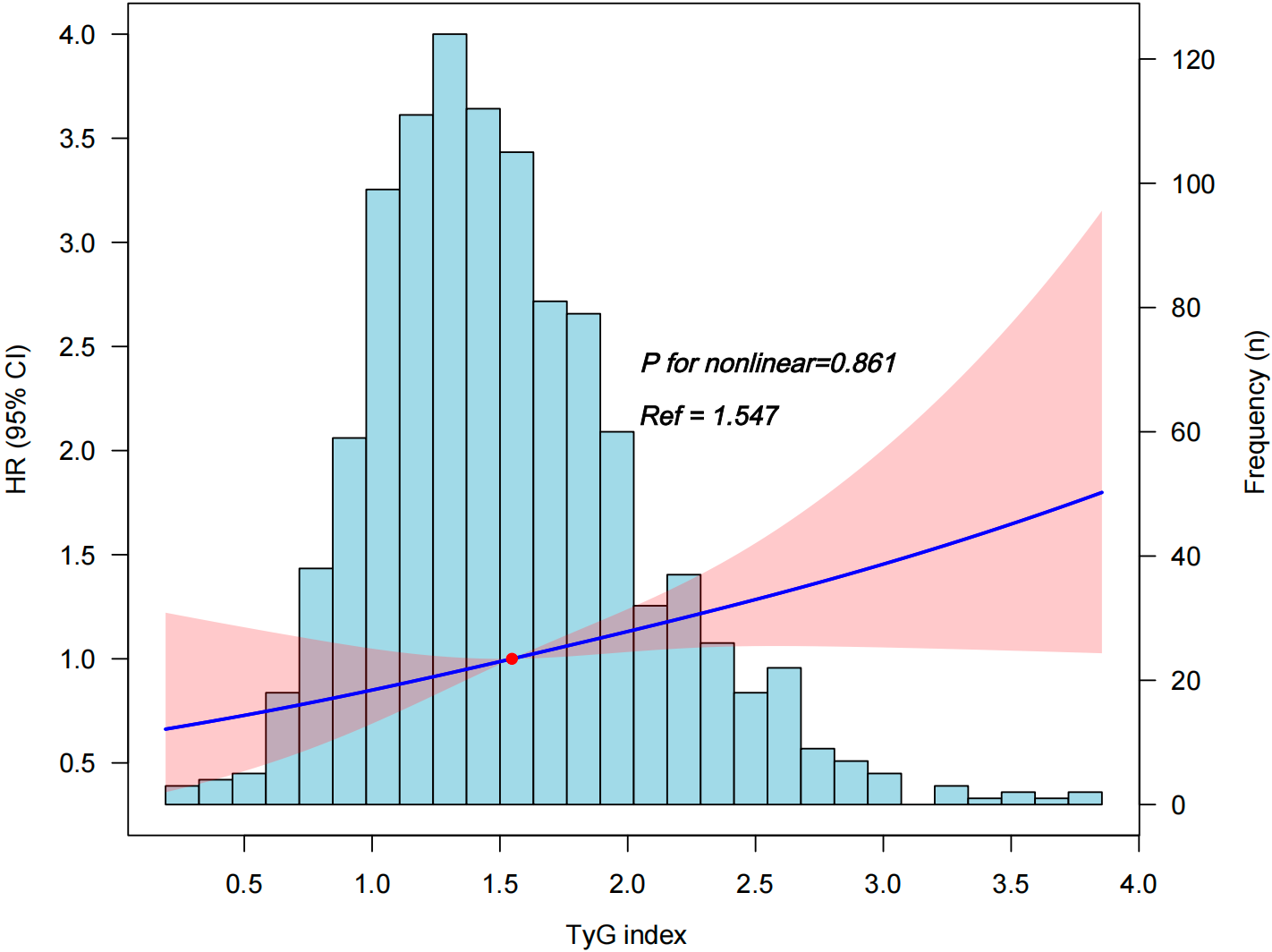
**

**Fig.S2** After adjustment for age, gender, race, hypertension, diabetes, stroke, dyslipidemia, myocardial infarction, atrial fibrillation, chronic kidney disease, acute kidney injury, chronic obstructive pulmonary disease, respiratory failure, the RCS curves of SHR and TyG index with mortality in HF patients. **A** and **B**, 90-day; **C** and **D**, 30-day.

**A B**


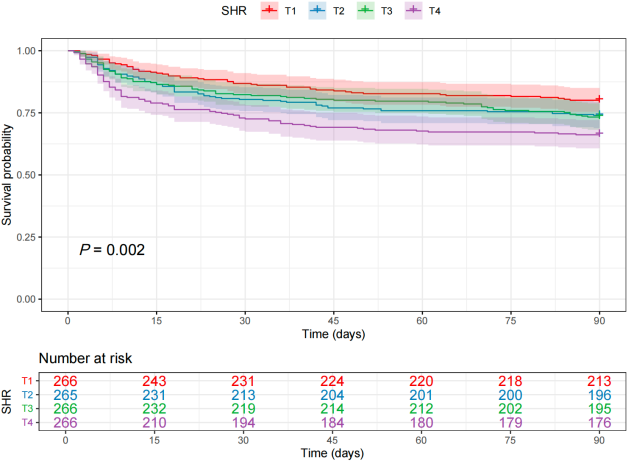

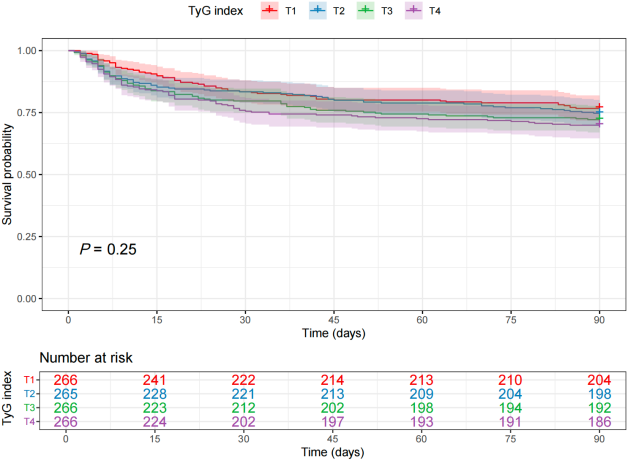


**C D**


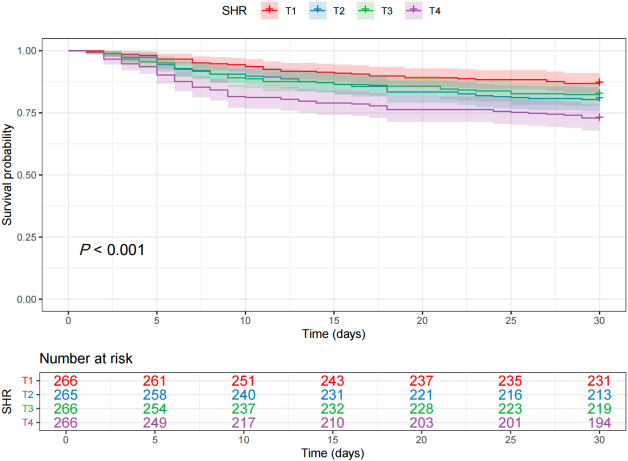

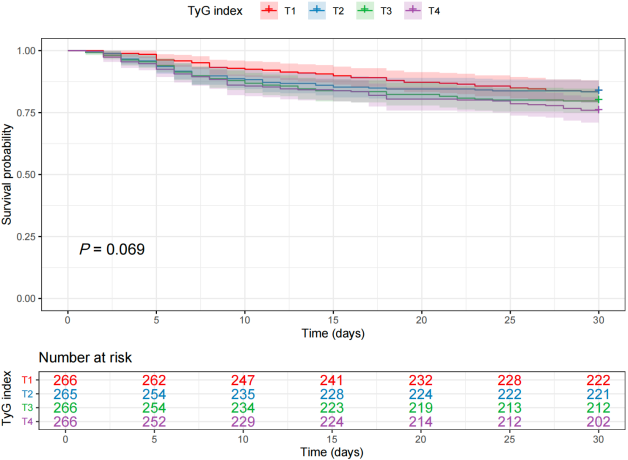


**Fig.S3** K-M curves of SHR and TyG index with mortality in HF patients, stratified by quartiles. **A** and **B**, 90-day; **C** and **D**, 30-day.

**A B**


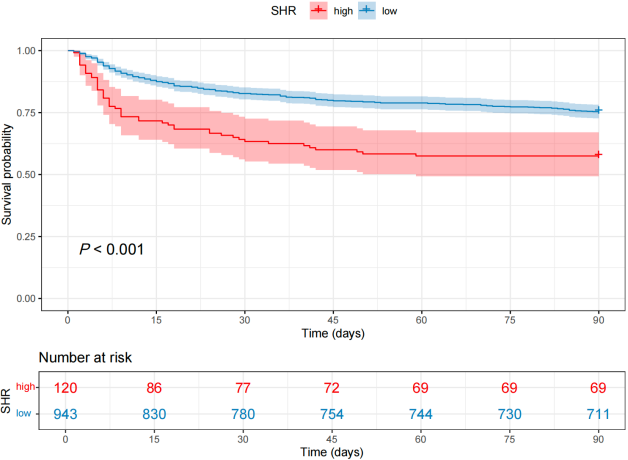

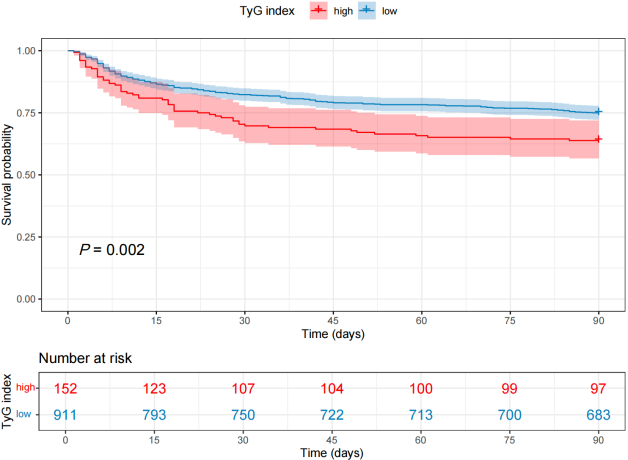


**C D**


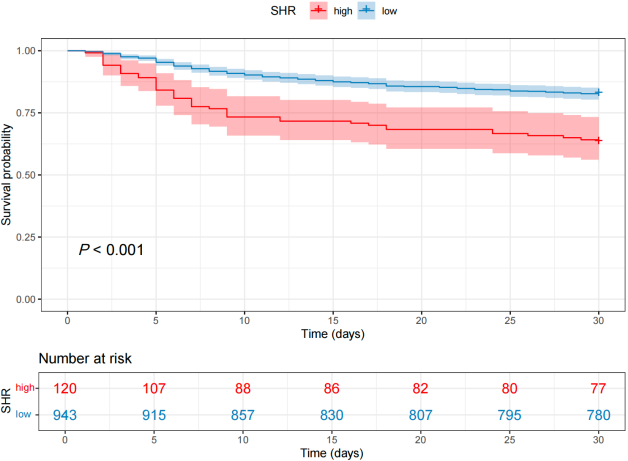

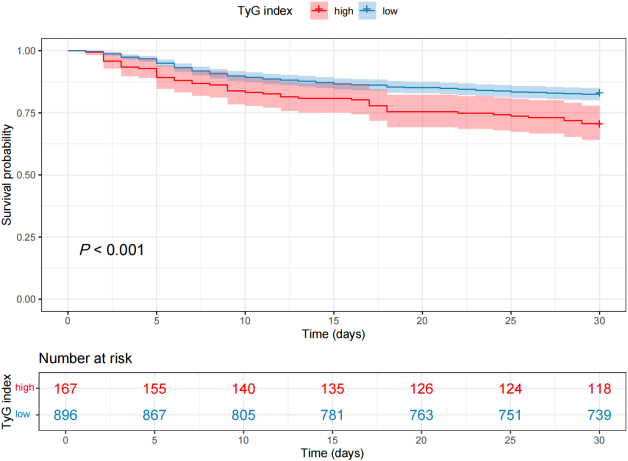


**Fig.S4** K-M curves of SHR and TyG index with mortality in HF patients, dichotomized by optimal cutoffs. **A** and **B**, 90-day; **C** and **D**, 30-day.

A B


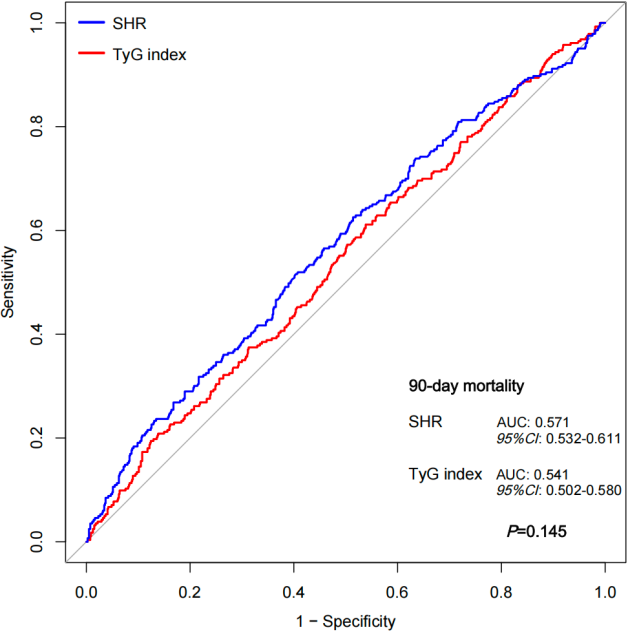

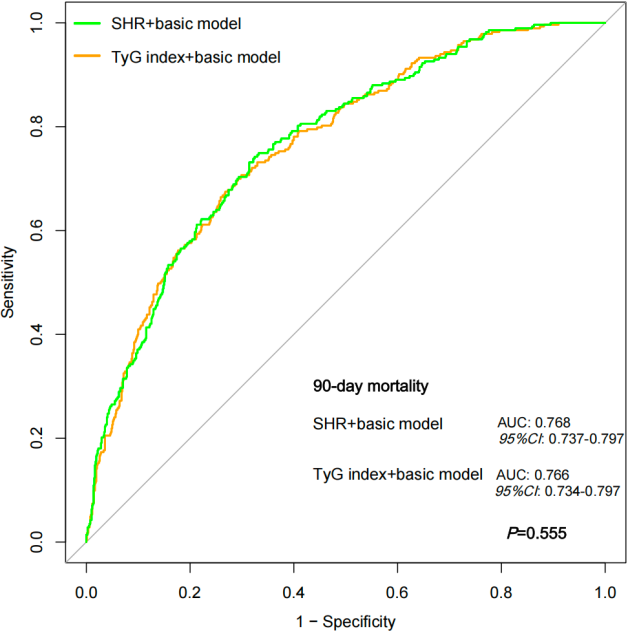


C D


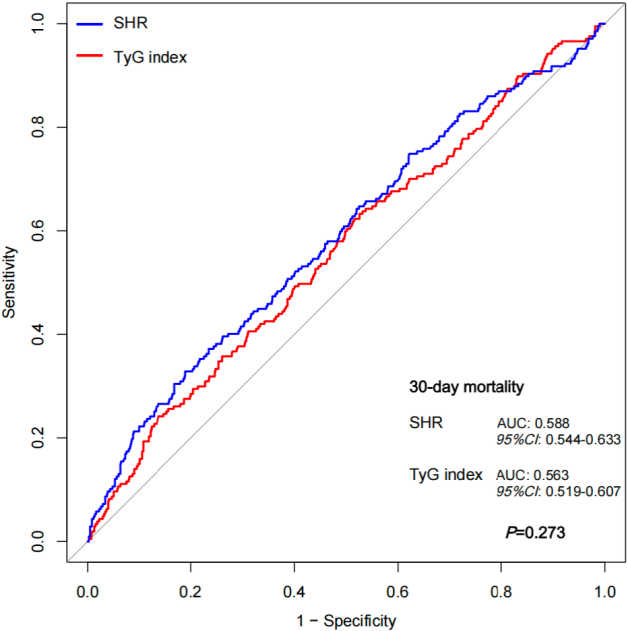

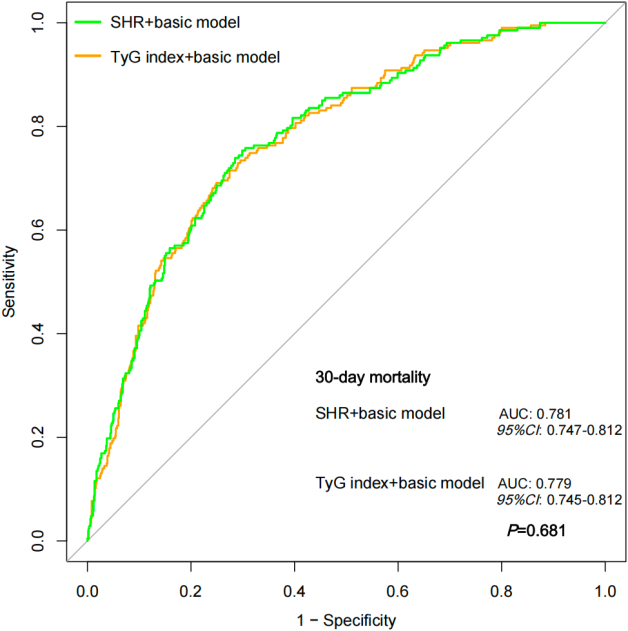


**Fig.S5** ROC curves of TyG index and SHR for predicting mortality in HF patients. **A** and **B**, 90-day; **C** and **D**, 30-day.

**A**


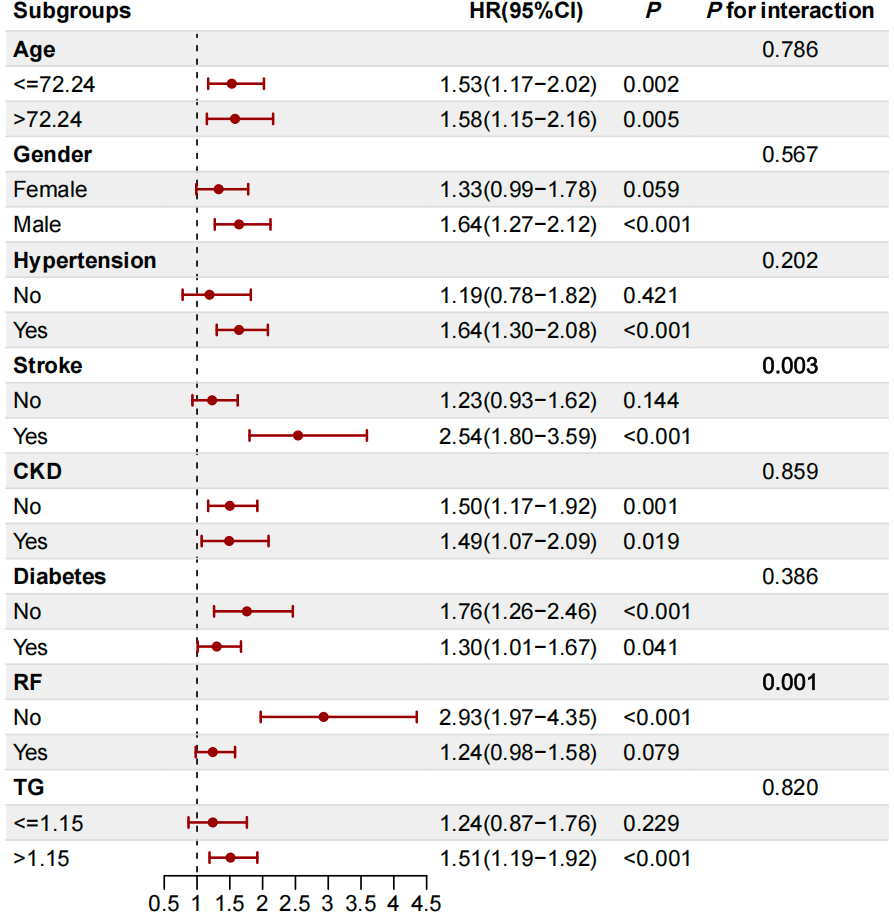


**B**


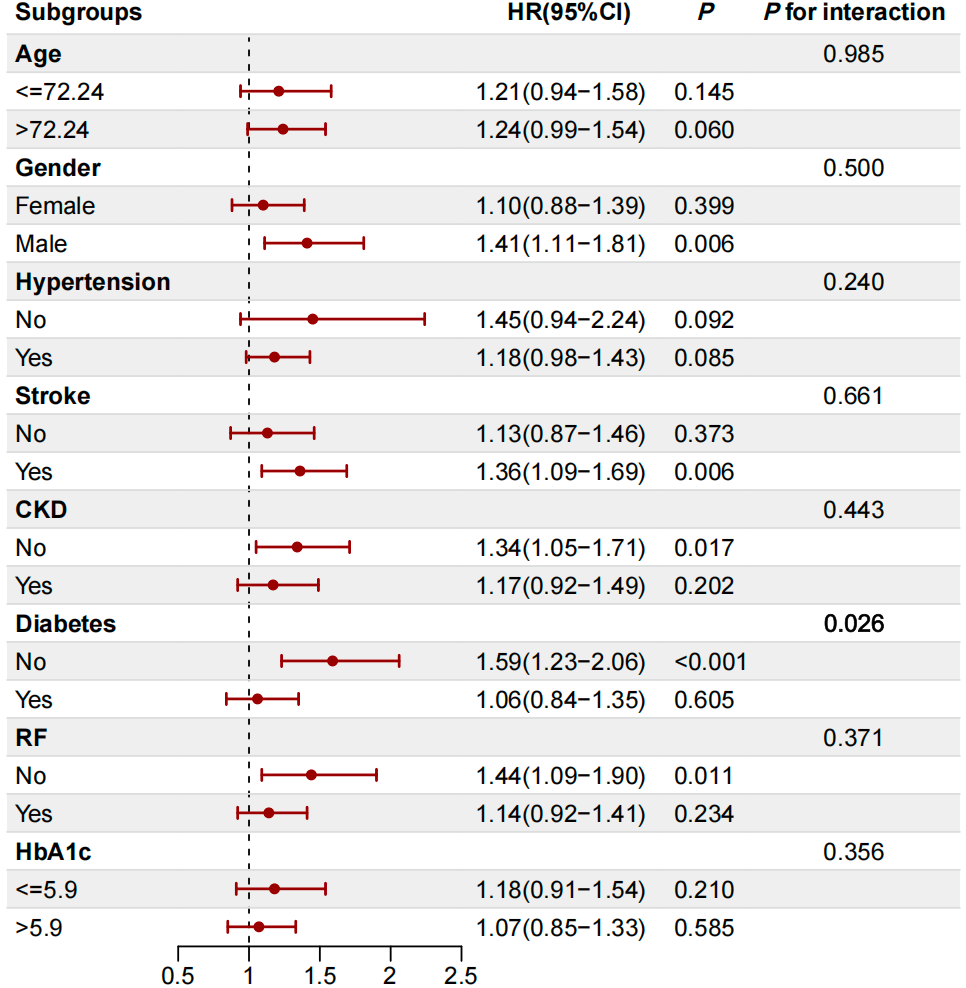


**Fig.S6** Associations of SHR and TyG index with 90-day mortality in HF patients by subgroups. A, SHR; B, TyG index.

**A**


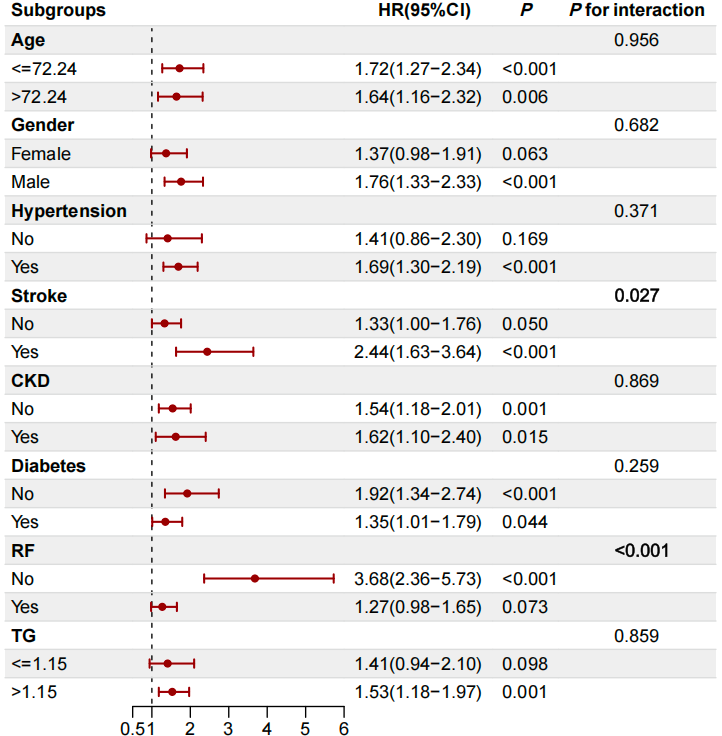


**B**


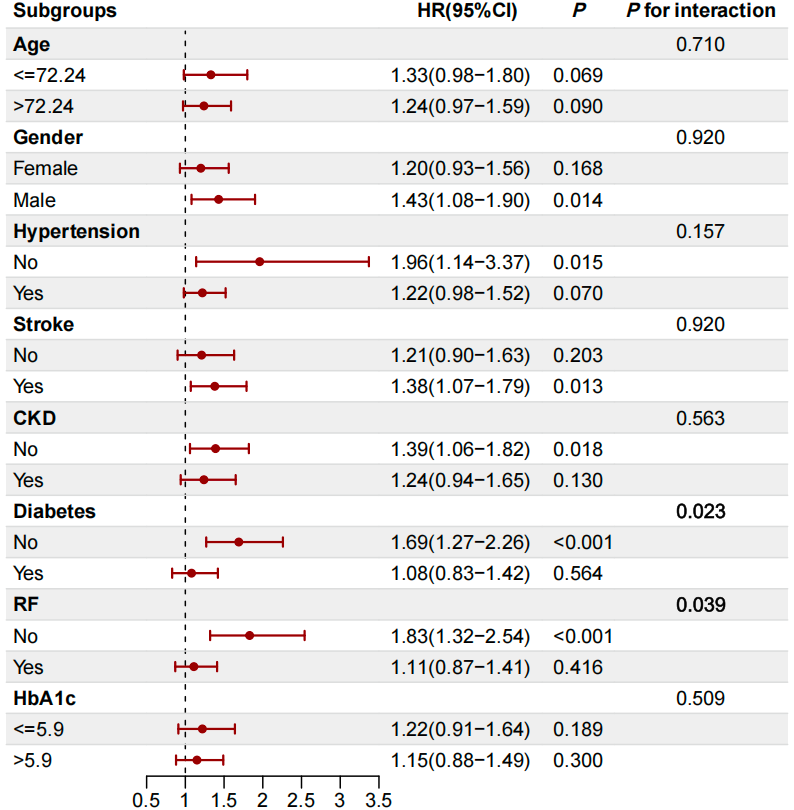


**Fig.S7** Associations of SHR and TyG index with 30-day mortality in HF patients by subgroups. A, SHR; B, TyG index.
