# Supplementary material for: Head-to-head comparison of stress hyperglycemia ratio versus triglyceride-glucose index for predicting mortality in heart failure: a retrospective cohort study
Source: Front Endocrinol (Lausanne). 2026 Feb 12;17:1782922. doi: 10.3389/fendo.2026.1782922 (PMC12935658; doi:10.3389/fendo.2026.1782922)
Supplement: Supplementary file 1 [file DataSheet1.zip › supplementary material/Supplementary Material 1.docx]

| icd_version | icd_code | long_title |
| --- | --- | --- |
| 9 | 39891 | Rheumatic heart failure (congestive) |
|  | 40201 | Malignant hypertensive heart disease with heart failure |
|  | 40211 | Benign hypertensive heart disease with heart failure |
|  | 40291 | Unspecified hypertensive heart disease with heart failure |
|  | 40401 | Hypertensive heart and chronic kidney disease, malignant, with heart failure and with chronic kidney disease stage I through stage IV, or unspecified |
|  | 40403 | Hypertensive heart and chronic kidney disease, malignant, with heart failure and with chronic kidney disease stage V or end stage renal disease |
|  | 40411 | Hypertensive heart and chronic kidney disease, benign, with heart failure and with chronic kidney disease stage I through stage IV, or unspecified |
|  | 40413 | Hypertensive heart and chronic kidney disease, benign, with heart failure and chronic kidney disease stage V or end stage renal disease |
|  | 40491 | Hypertensive heart and chronic kidney disease, unspecified, with heart failure and with chronic kidney disease stage I through stage IV, or unspecified |
|  | 40493 | Hypertensive heart and chronic kidney disease, unspecified, with heart failure and chronic kidney disease stage V or end stage renal disease |
|  | 4280 | Congestive heart failure, unspecified |
|  | 4281 | Left heart failure |
|  | 42820 | Systolic heart failure, unspecified |
|  | 42821 | Acute systolic heart failure |
|  | 42822 | Chronic systolic heart failure |
|  | 42823 | Acute on chronic systolic heart failure |
|  | 42830 | Diastolic heart failure, unspecified |
|  | 42831 | Acute diastolic heart failure |
|  | 42832 | Chronic diastolic heart failure |
|  | 42833 | Acute on chronic diastolic heart failure |
|  | 42840 | Combined systolic and diastolic heart failure, unspecified |
|  | 42841 | Acute combined systolic and diastolic heart failure |
|  | 42842 | Chronic combined systolic and diastolic heart failure |
|  | 42843 | Acute on chronic combined systolic and diastolic heart failure |
|  | 4289 | Heart failure, unspecified |
| 10 | I0981 | Rheumatic heart failure |
|  | I110 | Hypertensive heart disease with heart failure |
|  | I130 | Hypertensive heart and chronic kidney disease with heart failure and stage 1 through stage 4 chronic kidney disease, or unspecified chronic kidney disease |
|  | I132 | Hypertensive heart and chronic kidney disease with heart failure and with stage 5 chronic kidney disease, or end stage renal disease |
|  | I50 | Heart failure |
|  | I501 | Left ventricular failure, unspecified |
|  | I502 | Systolic (congestive) heart failure |
|  | I5020 | Unspecified systolic (congestive) heart failure |
|  | I5021 | Acute systolic (congestive) heart failure |
|  | I5022 | Chronic systolic (congestive) heart failure |
|  | I5023 | Acute on chronic systolic (congestive) heart failure |
|  | I503 | Diastolic (congestive) heart failure |
|  | I5030 | Unspecified diastolic (congestive) heart failure |
|  | I5031 | Acute diastolic (congestive) heart failure |
|  | I5032 | Chronic diastolic (congestive) heart failure |
|  | I5033 | Acute on chronic diastolic (congestive) heart failure |
|  | I504 | Combined systolic (congestive) and diastolic (congestive) heart failure |
|  | I5040 | Unspecified combined systolic (congestive) and diastolic (congestive) heart failure |
|  | I5041 | Acute combined systolic (congestive) and diastolic (congestive) heart failure |
|  | I5042 | Chronic combined systolic (congestive) and diastolic (congestive) heart failure |
|  | I5043 | Acute on chronic combined systolic (congestive) and diastolic (congestive) heart failure |
|  | I508 | Other heart failure |
|  | I5081 | Right heart failure |
|  | I50810 | Right heart failure, unspecified |
|  | I50811 | Acute right heart failure |
|  | I50812 | Chronic right heart failure |
|  | I50813 | Acute on chronic right heart failure |
|  | I50814 | Right heart failure due to left heart failure |
|  | I5082 | Biventricular heart failure |
|  | I5083 | High output heart failure |
|  | I5084 | End stage heart failure |
|  | I5089 | Other heart failure |
|  | I509 | Heart failure, unspecified |
|  | I9713 | Postprocedural heart failure |
|  | I97130 | Postprocedural heart failure following cardiac surgery |
|  | I97131 | Postprocedural heart failure following other surgery |
